# X‐ray micro‐CT imaging to study foliar water uptake mechanisms in plants with contrasting leaf topography

**DOI:** 10.1111/nph.70421

**Published:** 2025-08-09

**Authors:** Max Frank, Emil Visby Kristensen, Augusta Szameitat, Francesca Siracusa, Idil Ertem, Rikke Dahl, Katie G. Dempsey, Rajmund Mokso, Søren Husted

**Affiliations:** ^1^ Department of Plant and Environmental Sciences (PLEN) University of Copenhagen (KU) Thorvaldsensvej 40 1871 Frederiksberg C Denmark; ^2^ Department of Physics Technical University of Denmark (DTU) Fysikvej Building 310 2800 Kgs. Lyngby Denmark

**Keywords:** foliar water film, foliar water uptake, hydraulic activation of stomata, leaf topography, leaf wetness, stomata, X‐ray micro CT

## Abstract

- Formation of an aqueous continuum from the leaf surface to the sub‐stomatal cavity is a key process, affecting the foliar entry of solutes, particles, and pathogens. However, the factors controlling the transition from a water droplet to the formation of a continuous water film remain poorly understood.
- To address current limitations in state‐of‐the‐art bioimaging methods, we developed an X‐ray micro‐CT technique that enables nondestructive, time‐resolved visualization of water films on live barley (*Hordeum vulgare*) and potato (*Solanum tuberosum*) plants under controlled environmental conditions.
- We compare droplet behavior, leaf wetting, and the formation of foliar water films on two important crop plants, potato and barley, which differ markedly in their leaf surface characteristics, in terms of hydrophobicity of the cuticle, as well as stomatal topography and trichome morphology and density.
- We show that continuous water films, from the cuticle into stomata, may form within a few hours, and that a given set of environmental conditions may trigger hydraulic activation of stomata in one crop but not in another, depending largely on the physicochemical properties of the liquid and leaf surface morphological features.

## Introduction

Throughout the course of the last decades, plant scientists have become more and more intrigued by the fact that most plant species are apparently capable of absorbing liquid water through the leaves. Foliar water uptake (FWU) occurs across the leaf cuticle, or via surface structures such as hydathodes, trichomes, and stomata (Fernández *et al*., 2016; Berry *et al*., 2019; Schreel & Steppe, 2020; Schreel *et al*., 2020; Guzmán‐Delgado *et al*., 2021). The contribution of each individual assimilation pathway to the fate of the droplet will depend largely on environmental conditions, as well as the properties of the liquid and the leaf. The fate of a droplet, in turn, has strong implications for drought coping strategies (Schreel *et al*., 2022), potential pathogen infections (Velásquez *et al*., 2018), or foliar aerosol, nutrient, and pesticide absorption (Basi *et al*., 2014; Burkhardt *et al*., 2018; Arsic *et al*., 2022).

For a long time, both plant cuticles and stomata have been considered impermeable to water molecules and hydrophilic solutes. It was only in the 2000s that evidence of solute transport across isolated cuticles challenged this paradigm, and ‘hydrophilic pores’ were used as a term to describe these observations (Schönherr, 2000; Eichert & Goldbach, 2008; Fernández & Eichert, 2009). The model was refined in the 2010s, when TEM and AFM images indicated that cuticles were not just the hydrophobic layer of waxes, as previously described. Rather, they appear to be three‐dimensional scaffolds of carbohydrate chains emanating from the primary cell wall of epidermal cells (Fernández *et al*., 2016). Toward the leaf surface, these are increasingly enriched in waxes, cutin, and sometimes pectin. At high humidity, cuticles can swell as carbohydrates sequester water molecules, giving rise to ‘transient hydrophilic pores’ (Fernández *et al*., 2016, 2017; Tredenick & Farquhar, 2021). Today, we believe that these pores are a major cuticular entrance pathway for water, mineral ions, and other hydrophilic compounds below a size of *c*. 5 nm (Fernández *et al*., 2021; Arsic *et al*., 2022; Eichert & Fernández, 2023; Husted *et al*., 2023). However, little attention has been paid to the diversity of plant cuticles, which can vary in thickness and chemical composition at the submicron level within a single leaf (Fernández *et al*., 2024), as well as across plant species, age, leaf side, and the conditions to which plants have been exposed (Fernández *et al*., 2014). The current understanding of cuticular water uptake is vastly based on indirect observations due to a lack of appropriate bioimaging methods that allow tracing water molecules across cuticles (Fernández *et al*., 2021).

Similarly, stomata have been considered impenetrable for aqueous solutions for decades, due to the strong hydrophobicity of guard cell surfaces and their capillary resistance related to a sophisticated architecture (Schönherr & Bukovac, 1972). About 15 yr ago, Jürgen Burkhardt became aware that this barrier can be crossed in cases where a thin water film forms on the surface of stomatal guard cells, establishing hydraulic connectivity between the leaf surface and the sub‐stomatal cavity. This process was termed ‘hydraulic activation of stomata’ (HAS) (Burkhardt, 2010; Burkhardt *et al*., 2012). In this paper, we utilize the term ‘aqueous continuum’ to describe the thin layer of water that stretches from the leaf surface into the stomatal pore. Stomatal aperture and high relative humidity have been reported to be the major prerequisites for stomatal water uptake in the form of water vapor condensing inside stomatal cavities (Berry *et al*., 2014; Guzmán‐Delgado *et al*., 2021). By contrast, the uptake of liquid water, solutes, and particles across stomatal pores is believed to exclusively occur upon HAS (Burkhardt *et al*., 2012; Basi *et al*., 2014). HAS formation is the result of deliquescence of hygroscopic particles in the presence of humidity (Burkhardt *et al*., 2012; Basi *et al*., 2014). Whether stomatal aperture is a prerequisite for HAS remains to be verified. However, once HAS has been established, it appears to prevail to some extent after stomatal closure (Vega *et al*., 2023). Leaf wetting agents, such as surfactants, may also assist HAS formation (Kaiser, 2014; Fernández *et al*., 2021). The literature suggests that this is achieved by overcoming the capillary resistance of stomata at liquid surface tensions below a threshold of *c*. 30 mN m^−1^ (Schönherr & Bukovac, 1972). Based on a few experiments, in which penetration by particles and ions was found in < 10% of the stomatal pores present on the leaf surface, calculations estimated that the intrastomatal water continuum is < 100 nm thick (Eichert & Burkhardt, 2001; Burkhardt *et al*., 2012). The assumption that only a fraction of all stomata on a leaf is activated simultaneously seemed to be supported by further observations (Vega *et al*., 2023) and has been repeated in publications without being experimentally challenged (Schreel *et al*., 2020; Eichert & Fernández, 2023; Husted *et al*., 2023). However, similar to cuticular transport, HAS is a model based on indirect evidence from transport of solutes and particles (Eichert *et al*., 2008; Eichert & Goldbach, 2008; Burkhardt *et al*., 2012, 2018; Basi *et al*., 2014), stable isotope mass balances (Goldsmith *et al*., 2017), gravimetric measurements (Vega *et al*., 2021, 2023), and observations of stomatal leakiness (Grantz *et al*., 2018; Vega *et al*., 2023). The conditions that lead to the formation of stomatal water continuums, their thickness, and their quantitative occurrence in different plant species under varying forms of leaf wetness remain a major research gap.

For clarity in this work, we define a water droplet on a leaf surface as a spatially confined unit with a defined contact angle. In this context, a water film on a leaf is the result of a droplet that spreads out over an undefined leaf surface area, resulting in a liquid layer of varying thickness in the low μm range that follows the leaf topography and does not have a specified contact angle. Whenever water originating from a droplet or film enters a stomatal pore, we speak of a continuum spanning into the sub‐stomatal cavity.

When we address the establishment of a direct connection between a droplet or liquid film on the leaf surface with the hydraulic system within the leaf tissue, spanning either cuticle or stomata, we choose to speak of the activation of the respective FWU pathway.

Because available imaging techniques have various limitations for tracing water films on leaf surfaces, we developed an X‐ray micro computed tomography (μCT)‐based imaging method. The method allows us to study foliar water film development on leaves of live plants in a controlled atmosphere over time. X‐ray μCT has increasingly been developed within plant science over the last two decades, and its application in plant science has been comprehensively reviewed (Dhondt *et al*., 2010; Chen *et al*., 2021; Piovesan *et al*., 2021). Existing studies address pore space analyses of plant tissues (Cloetens *et al*., 2006; Herremans *et al*., 2015; Chen *et al*., 2021; Gao *et al*., 2023), water and solute transport in roots and stems (Cochard *et al*., 2015; Scotson *et al*., 2021; Hou *et al*., 2022; Schreel *et al*., 2022; Camboué *et al*., 2024), water uptake through trichomes (Schreel *et al*., 2020), or quantification of crystalline structures (Earles *et al*., 2018). Most studies have been conducted on dead plant material (i.e. wood) and rigid live tissues (e.g. roots, vines, and conifer needles) (Chen *et al*., 2021; Scotson *et al*., 2021; Hou *et al*., 2022; Gao *et al*., 2023), or detached organs (e.g. buds, fruits, seeds, or leaves) (Cloetens *et al*., 2006; Verboven *et al*., 2015; Mathers *et al*., 2018; Duncan *et al*., 2022). Few studies have been conducted on soft foliar tissues of live plants at the μm resolution scale (Piovesan *et al*., 2021); however, to the best of our knowledge, no X‐ray μCT studies have been conducted within a controlled environment. In this study, we compare leaf wetting and FWU dynamics into leaves of two important crop plants, potato and barley, which differ markedly in their leaf surface characteristics. By forcing stomata open under high relative humidity, we create optimal conditions for the activation of the cuticular and stomatal pathways. By using the newly developed X‐ray μCT‐based imaging methodology, we hypothesize the following:

Once a droplet is deposited on a leaf surface, a wettable leaf surface will promote the conversion of a defined droplet into a water film, even at relatively high droplet surface tensions, while nonwettable leaf surfaces will require low surface tensions to allow for water film formation. We show the role of leaf topography and droplet surface tension in determining the extent of leaf surface contact with overlying liquid. As a consequence, the preferred FWU pathways will differ between leaves of barley and potato plants, as these vary in their surface wettability, topography, and stomatal and trichome morphology. Because these features differ markedly among plant species, we show that a specific surface tension might result in the activation of the cuticular or stomatal pathway in one species, while not activating these pathways to the same extent in a different species. In contrast to the existing literature on the subject, we show that stomata can become hydraulically activated within a few hours in some species, even at high surface tension of droplets, provided that they are open and the relative air humidity is high.

We believe that the method presented in this paper will pave the way for a wide variety of future experiments where live plants can be imaged while being exposed to external stimuli such as heat, drought, toxins, or pathogens.

## Materials and Methods

### Plant cultivation

Barley, *Hordeum vulgare* L. cv KWS Thallis was germinated in vermiculite 14 d before the onset of the experiments (glasshouse: 16 h : 8 h, 19°C : 15°C, day : night, RH = 70%, PPFD = 300 μmol m^−2^ s^−1^). When germinated, seedlings were singularized. Potato, *Solanum tuberosum* L. cv Wotan, cuttings were produced from mother plants 12 d before the experiment started and rooted in vermiculite (glasshouse: 16 h : 8 h, 23°C : 18°C, day : night, PPFD = 200 μmol m^−2^ s^−1^). All plants received nutrient solution after 10 d. The composition of the nutrient solution for barley is described in Arsic *et al*. (2022). However, potato plants were grown with a solution containing double the reported nutrient concentrations.

### Leaf surface wetting dynamics

Youngest fully evolved leaves of potato and barley plants were fixed onto a glass slide with double‐sided tape. A heptamethyltrisiloxane‐based superspreader (Silwet Gold (HD 2412, DK)) was used in order to adjust the surface tension of water to γ = 20, 30, 40, 50, 60, and 70 mN m^−1^ using 0.050, 0.010, 0.005, 0.003, 0.001, and 0.000% w/v Silwet Gold, respectively (Supporting Information Fig. S1). Droplets of 10–20 μl volume were deposited on the adaxial leaf side. Spreading and dynamics were followed over time with an optical tensiometer (ThetaFlow; Biolin Scientific, Gothenburg, Sweden) at 21°C and 20% RH.

### Stomatal conductance measurements

Stomatal opening was investigated by controlling light and CO_2_ concentration in a leaf cuvette of a CIRAS 2 H_2_O/CO_2_ gas analyzer (PP Systems, Amesbury, MA, USA). Once a leaf was placed in the cuvette without light, the gas composition was allowed to stabilize at ambient CO_2_, and plants were given 30 min to adapt. Then, stomatal conductance (*g*_s_) was monitored. Stabilization of *g*_s_ measurements, followed by stepwise adjustments of cuvette conditions, was as follows: lowering CO_2_ to 50 ppm, illumination with 390 PAR, and finally switching off the light with CO_2_ readjusted to 400 ppm.

### Leaf epidermal imprints and low CO_2_

Potato and barley plants were placed in a gas‐tight Plexiglas chamber under 390 PAR. Dry air was passed through Plexiglas columns (PP Systems) of soda lime ACS (Thermo Scientific, Waltham, MA, USA) to obtain < 50 ppm CO_2_. The CO_2_‐reduced air was guided through a series of two 1 l water‐filled impinger flasks (Lenz, Wertheim, Germany) to achieve > 90% RH. CO_2_ and RH were monitored with a CIRAS‐2 H_2_O/CO_2_ gas analyzer (PP Systems) at the exit of the chamber. Once measurements had stablilized, plants were allowed to adapt for 30 min. The chamber was then opened, and a thin film of transparent nail polish (Essie, Astoria, NY, USA) was applied to the leaf surface. The nail polish was allowed to dry, and the leaf epidermal imprint was imaged with a Leica DM2000 LED, with ×10/0.25 objective lens and a Flexcam C1 camera (Leica Microsystems GmbH, Wetzlar, Germany). Because epidermal imprints of potato leaves were not sufficiently planar to create good image quality, focused images were computed from z‐stacks of focal planes by using Helicon Focus 8 (Helicon Soft Ltd, 2000, Charkiw, Ukraine) with standard settings.

### Plant handling for X‐ray μCT scans

Plant handling was carried out in a laboratory adjacent to the DANMAX beamline hutch at the MAX IV synchrotron in Lund, Sweden. In order to force stomatal aperture, living plants were placed in the sample holder with their roots placed in vermiculite soaked with nutrient solution. The sample holder is described in detail in the Results section. An intact young leaf was gently fixed onto the ‘wing’ by using double‐sided tape and Parafilm® (VWR, Radnor, PA, USA) in order to avoid any movement during the CT scan. Air with < 50 ppm CO_2_ and > 90% RH was passed through the closed chamber as described in the section ‘Leaf epidermal imprints and low CO_2_’. After 30 min, 20 μl droplets of 150 mM iohexol (VWR) at surface tensions of 20, 30, 40, and 70 mN m^−1^ were placed on the leaf surface. The surface tension of the droplets was controlled by using 0.03%, 0.01%, 0.005%, and 0% Silwet Gold, respectively (Fig. S1). The chamber was closed again and the plants remained in the controlled atmosphere until the CT scan. For the CT scan, the gas tubing was removed from the sample holder. Inlet and outlet were sealed, and the sample holder was fixed on the sample stage. Once scanned, the sample holder with the plant was placed back into the tubing line until the next scan was conducted. Preliminary tests showed that the gas composition in the sealed sample holders stayed constant over > 22 h (data not shown), and stomata were forced to open (Figs S2–S6). Plants were scanned repeatedly in different leaf areas over time.

### X‐ray μCT scans

Before CT scanning, samples were transferred in the sealed sample holder from the laboratory to the rotational stage in the X‐ray hutch. During measurements, samples were exposed to 390 PAR sustaining stomatal aperture. The monochromator was set to 35 keV X‐ray energy to stay above the iodine Kα edge at 33.2 keV. This high energy also ensured low radiation damage to the plant samples due to the low absorption and high phase contrast at higher energies. In this configuration, the beam FWHM size is 1.2 × 1.3 mm^2^ with a total flux of *c*. 5 × 10^12^ ph s^−1^ at the sample position. For optimal phase contrast by single‐distance phase retrieval, the sample was placed as close to the detector as allowed by the sample holder, giving a sample to detector propagation distance of *c*. 70 mm. For X‐ray detection, an indirect method was used with a scintillator screen, a ×20 objective, and a 4608 × 2592 pixels Orca Lightning Hamamatsu camera, giving a pixel size of 0.275 μm. For faster collection, the camera was configured for a low dynamic range of 12 bits. To ensure a minimum of radiation damage, samples were aligned to the region of interest using a set of filters reducing the flux by a factor of 79. Before each scan, flat and dark field images were collected to correct for beam shape. Over 360°, 3001 projections were collected with an exposure time of 3 ms for each projection.

### Data processing and image reconstruction

All projection image processing and 3D data reconstruction were performed using TomoPy (Gürsoy *et al*., 2014). Raw projections were normalized with flat‐field and dark‐field images and binned by a factor of 2, resulting in a 550 nm pixel size. Horizontal stripes in the sinograms were removed using the Fourier–Wavelet (FW) method (Mallat, 1999), employing the wavelet filter ‘sym5’ and a Fourier space damping parameter of σ = 2, as implemented in TomoPy. Phase retrieval using Paganin's single‐distance method (Paganin *et al*., 2002) was then applied, with a regularization parameter of α = 10^−4^ for barley leaves and α = 3 × 10^−4^ for potato samples. The center of rotation for each scan was found using using Nghia Vo's method (Vo *et al*., 2014). To minimize gray‐level variation artifacts caused by the strongly absorbing iohexol on leaf surfaces, the projections were padded at the sinogram edges using a tapering function, 1 − tanh(*x*), smoothly dropping from the edge value to zero. This padding ensured that all sinograms ended with zero values at the edges, preventing inconsistencies in tomogram intensity as a function of height for similar materials. Finally, the data were converted to −log(data) and reconstructed using a Fourier grid reconstruction algorithm. Post processing of the 3D data sets were done in Dragonfly using the Man3DRBF filter for shading correction and a median filter with kernel size 7 for image denoising.

## Results

### Droplet deposition and spreading

**Fig. 1:**
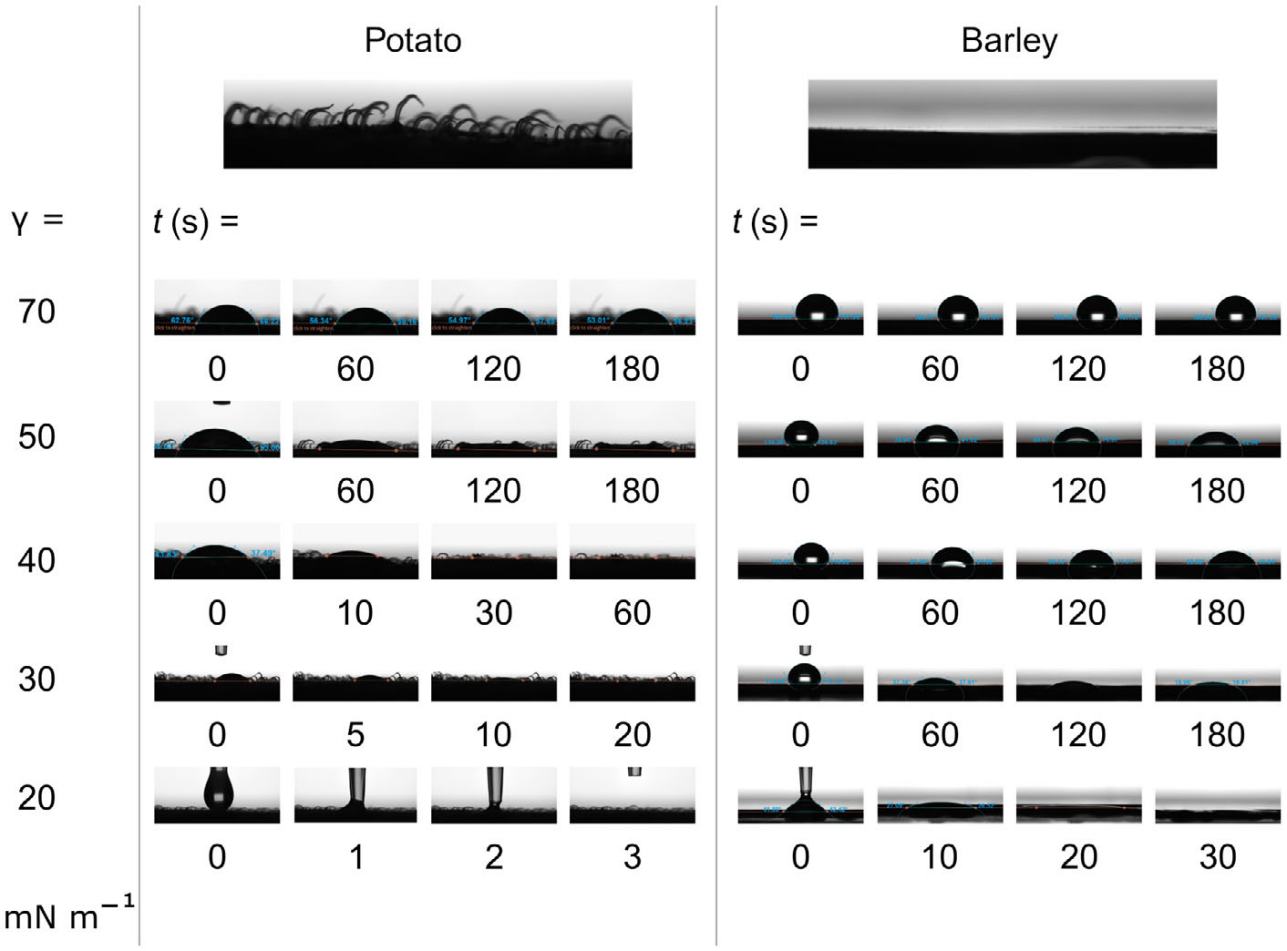
Leaf wetting dynamics. Images from an optical tensiometer showing droplet spreading on adaxial leaf surfaces of potato and barley at the indicated surface tensions over time. Droplet surface tensions were adjusted with the surfactant Silwet Gold.

First, we investigated the spreading of droplets on the adaxial side of youngest fully evolved leaves of 2‐wk‐old barley and potato plants. To this end, we used optical tensiometry and followed droplets with varying surface tensions on the leaves over time. The results are presented in Fig. 1. Potato leaves were uneven and covered with large trichomes. By contrast, barley leaves were more even, with few small trichomes located along parallel veins. Generally, leaves of potato plants were more wettable than those of barley plants. After 180 s, a droplet at the surface tension of water (γ = 70 mN m^−1^) began to visibly spread on a potato leaf, while it remained with a large contact angle on a barley leaf. As γ was lowered stepwise to 50, 40, 30, and 20 mN m^−1^, droplets spread out increasingly fast. Upon spreading, water films developed, covering a larger leaf area but decreasing in thickness of the film. A droplet at γ = 20 mN m^−1^ would spread out into a film instantaneously on a potato leaf, while still being distinct on a barley leaf after 10 s (Fig. 1).

Based on the literature, the cuticular and stomatal foliar uptake pathways both require a humid atmosphere, while the latter is also reported to be dependent on stomatal aperture. Therefore, we exposed potato and barley plants to a PPFD of 390 μmol m^−2^ s^−1^, > 90% RH, and < 50 ppm CO_2_. We measured stomatal conductance (*g*_s_) and confirmed full stomatal aperture by producing leaf epidermal imprints after plants had equilibrated to the atmosphere (Figs S2, S3).

**Fig. 2:**
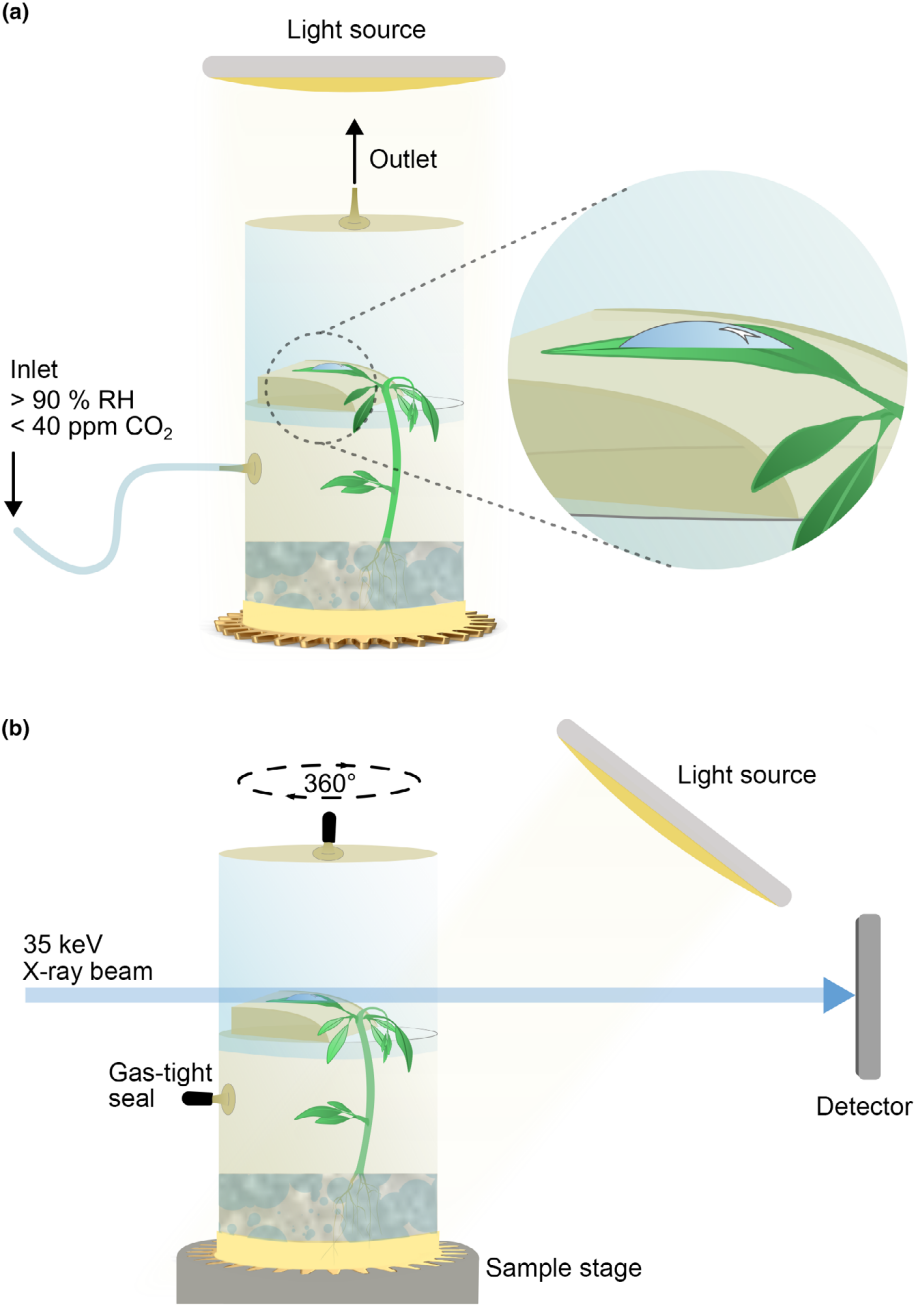
Sample holder and experimental setup. (a) A potato plant placed in the sample holder with the root space filled with vermiculite soaked with nutrient solution, and the leaf of interest fixed onto a plastic wing. A controlled gas flow passes through the sample chamber, and a light source provides suitable growth conditions. (b) When fixed onto the rotating sample stage at the beamline, the chamber was sealed to maintain the atmospheric conditions throughout the scanning time. After each scan, the plant was returned to the laboratory, as shown in (a), until the next scan.

The optical tensiometry data suggested that leaf wetting was a process that occurred on a time scale between seconds to minutes (Fig. 1), while previous studies suggest stomatal uptake to occur between hours and weeks (Burkhardt *et al*., 2012; Grantz *et al*., 2018; Avellan *et al*., 2019; Vega *et al*., 2023). For X‐ray μCT imaging of foliar water films, we therefore designed a gas‐tight plant growth chamber which allowed us to keep the growth conditions for barley and potato plants favorable over extended time periods (Fig. 2a). Simultaneously, the chamber served as a sample holder that could be easily mounted onto the CT sample stage (Fig. 2b). The growth chamber/sample holder was designed in a way that allowed us to overcome a row of technical constraints and challenges described in the following sections.

### Droplet persistence and air humidity

Generally, normal laboratory environments with low humidity, strong ventilation, and low light conditions lead to fast drying of droplets and films and might deactivate cuticular and stomatal uptake pathways. Therefore, the sample holder was continuously flushed via silicon tubings with the atmosphere described in the section ‘droplet deposition and spreading’. However, when mounted onto the sample stage, the sample holder is required to rotate by 360°. Having tubes attached during the CT scans would increase the risk of movements, which is critical as even sub‐μm scale vibrations will reduce image quality (unpublished preliminary work). Therefore, whenever a plant was CT scanned at the beamline, the sample holder could be detached from the tubing system and the atmosphere could be trapped by sealing in‐ and outlet with a rubber cap. Within a minute, it was possible to fix it on the sample stage and start the CT scan, where the plant was exposed to 390 PAR (Fig. 2b).

### Eliminating the effects of movement during CT scans

To eliminate the effect of movement during CT scanning, plants were grown in a similar atmosphere compared to the one they experienced in the beamline, reducing tissue stretching and bending in response to altered humidity, turgor, or light conditions (Fig. 2a,b). Secondly, the abaxial side of the scanned leaf was mounted on a 3D printed plastic ‘wing’ with double‐sided tape, and the petiole was kept as movement free as possible by fixing it to the wing with Parafilm® (Fig. 2a). Thirdly, scanning durations were limited to 9 s, reducing the time window for movements to occur. Including flat field and dark field collection, and motor movements, the total scan time was *c*. 3–4 min. A fourth measure was to 3D print a cogwheel‐shaped sample holder foot, which allowed direct transfer of the sample holder onto the sample stage, where a tight 3D printed fitting was mounted with screws (Fig. 2a,b). This fixed the sample holder within a few seconds and enabled vibration‐free scanning of the sample.

### Reducing tissue damage by X‐rays

To reduce radiation damage on the leaves, multiple parameters have been optimized. X‐ray phase contrast imaging was chosen due to higher dose efficiency for soft biological tissues compared to absorption‐based techniques (Spiecker *et al*., 2023).

For low‐density leaves, the optimal contrast‐to‐dose ratio was achieved by choosing a relatively high beam (30 keV < *E* < 40 keV), energy giving ample phase contrast while limiting damage from X‐ray absorption in the sample. This energy allows for ample X‐ray flux at the DANMAX beamline of MAX IV. While aligning the sample, an attenuation filter reduced the beam flux from 1.3 × 10^12^ ph s^−1^ mm^−2^ to *c*. 10^10^ ph s^−1^ mm^−2^. Finally, the exposure time for each projection was limited to 3 ms, providing enough signal for the analysis without overexposing the leaf to X‐rays. The estimated dose of a single scan under these conditions is calculated as:

$$D=\frac{I_{0}\cdot E\cdot t\cdot n}{A}\cdot \frac{\mu }{\rho }\approx 800\;\mathrm{Gy}$$

where $I_{0}$ is the incident flux, *E* the energy, *t* the exposure time, *n* the number of projections, *A* the area of the field of view, and $\frac{\mu }{\rho }$ the X‐ray mass attenuation coefficient. This dose level keeps well below the expected onset of radiation damage of *c*. 5 kGy (Jones *et al*., 2020). In our experiments, physical movement and water loss started after 7–10 scans and an accumulated dose of 5–8 kGy (data not shown).

### Contrast between applied water and tissue water

Based on an earlier study by Gao *et al*. (2023), where assimilate flows in apoplastic water were traced in pine needles by X‐ray μCT imaging, we used 150 mM iohexol as a water‐soluble contrast agent. The water film with iohexol was imaged with the monochromator of the X‐ray beam set to 35 keV, above the Kα edge of iodine at 33.2 keV. These settings allowed us to distinguish water films originating from deposited droplets from most foliar structures as well as from water within the tissue based on X‐ray absorption.

### Leaf surface morphology

Leaves of barley and potato plants, which were not exposed to a droplet, were used as a negative control that allowed studying the surface morphology and quantification of stomatal aperture.

**Fig. 3:**
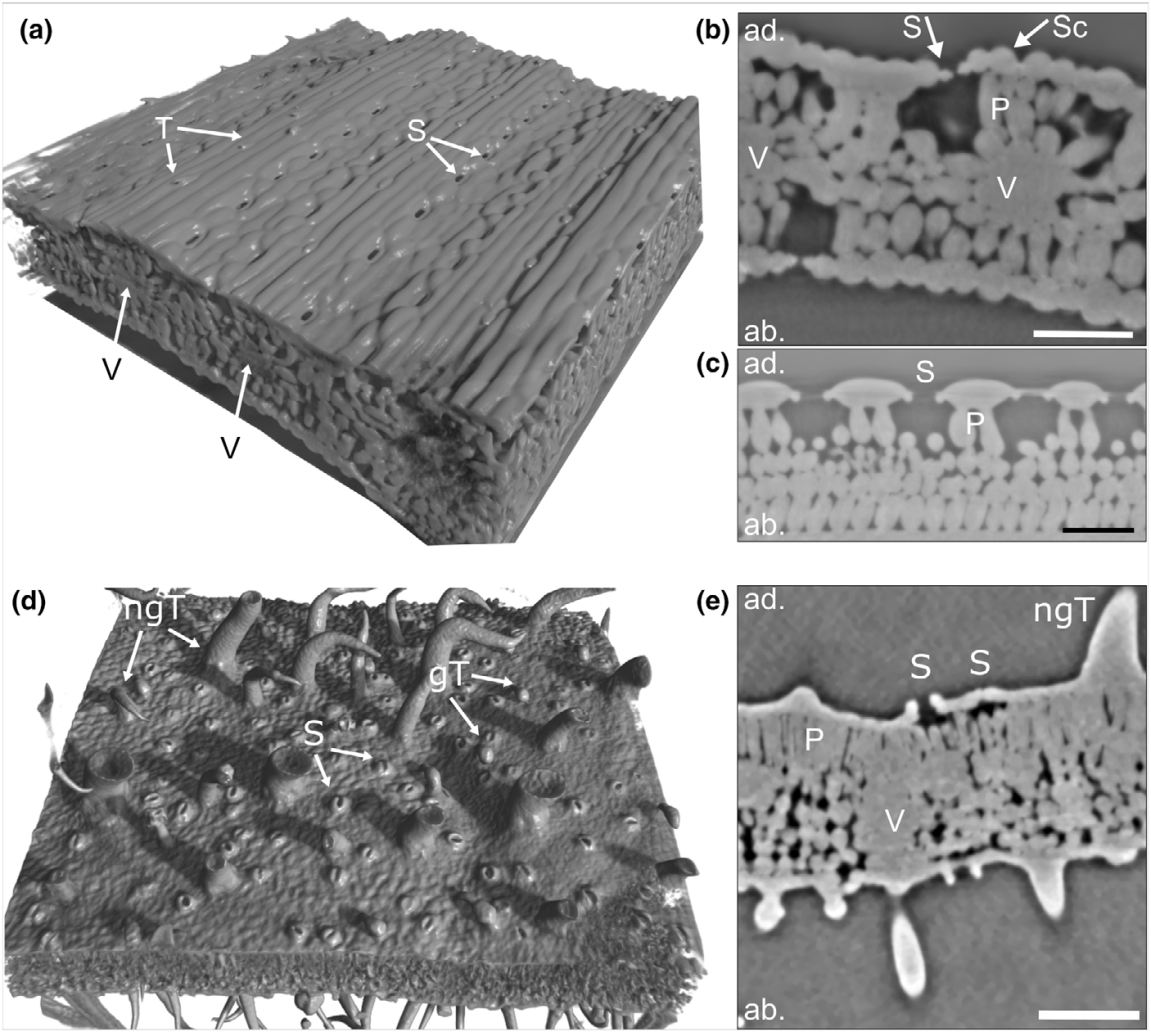
Morphology of barley and potato leaves. Reconstructions and virtual cross sections of untreated barley and potato leaves scanned in X‐ray μCT. (a–c) Barley, (d, e) potato, (a, d) adaxial leaf sides as 3D reconstructions. (b) Vertical cross section through a vein and an open stoma. (c) Vertical cross section along a row of open stomata. (e) Cross section through a vein, open stomata, and nonglandular trichomes. ab., abaxial leaf side; ad., adaxial leaf side; gT, glandular trichome; ngT, nonglandular trichome; P, palisades; S, stoma; Sc, sclerenchyma; T, trichome; V, vascular bundle. Bars, 100 μm.

The characteristics of barley leaves have been described in detail in Arsic *et al*. (2020). Barley leaves have parallel veins, which are topographically elevated (Fig. 3a; Video S1). Sclerenchymal tissue and nonglandular trichomes follow along these ridges (Fig. 3a,b; Video S1). Stomata are sunken structures, which are never located on the sclerenchymal ridges, but along parallel lines in the space between them (Fig. 3a–c; Video S1). In the absence of a droplet, no contrast was observed on the leaf surface or inside the tissue that resembled a water film (Fig. 3a–c; Video S1). At 390 PAR, > 90% RH and < 50 ppm CO_2_, 100% of the stomata were observed to be open. Stomatal apertures were measured between 7.7 and 12.7 μm, with an average aperture of 10.4 ± 0.15 μm (Figs S4, S6).

Potato leaf surfaces are described extensively by Papierowska *et al*. (2020). On potato leaves, veins are branched. Large nonglandular trichomes and smaller glandular trichomes are unevenly distributed on the adaxial leaf surface (Fig. 3d; Video S2). 87% of the stomata were observed to be open. The stomatal apertures ranged from entirely closed to an aperture of 28.6 μm with an average aperture width of 14.68 ± 0.93 μm (Figs 3d,e, S5, S6; Video S2). Fig. 3(e) shows that nonglandular trichome bases are enforced with sclerenchymatic cells. Veins generally appear as topographic depressions on the adaxial leaf side, while stomata are elevated above the surrounding leaf surface (Fig. 3d,e; Video S2). This property stands in contrast to the topography observed on barley leaves.

### The transition from droplet to water film

**Fig. 4:**
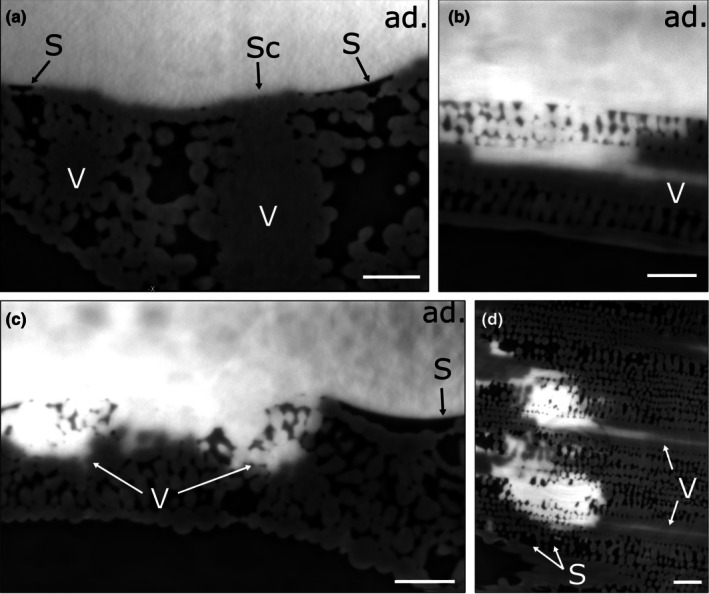
High surface tension droplets on a barley leaf. Virtual cross sections through a barley leaf with a 70 mN m^−1^ water droplet deposited on the adaxial leaf side and imaged by X‐ray μCT. Bright areas correspond to water deposited on the leaf surface, gray areas correspond to leaf tissue, and dark areas correspond to air space. (a) No water uptake into the leaf is observed 20 min after droplet deposition. (b–d) After 8 h, water uptake occurs across the cuticle, epidermal cells, and sclerenchyma rich in lignin and pectin into the vascular bundle, while most stomatal guard cell surfaces do not establish physical contact with the water droplet. (b) Cross‐section along a vein. (c) Cross‐section perpendicular to the veins. (d) Horizontal section through the leaf. ad, adaxial leaf side; S, stoma; Sc, sclerenchyma; V, vascular bundle. Bars, 100 μm.

**Fig. 5:**
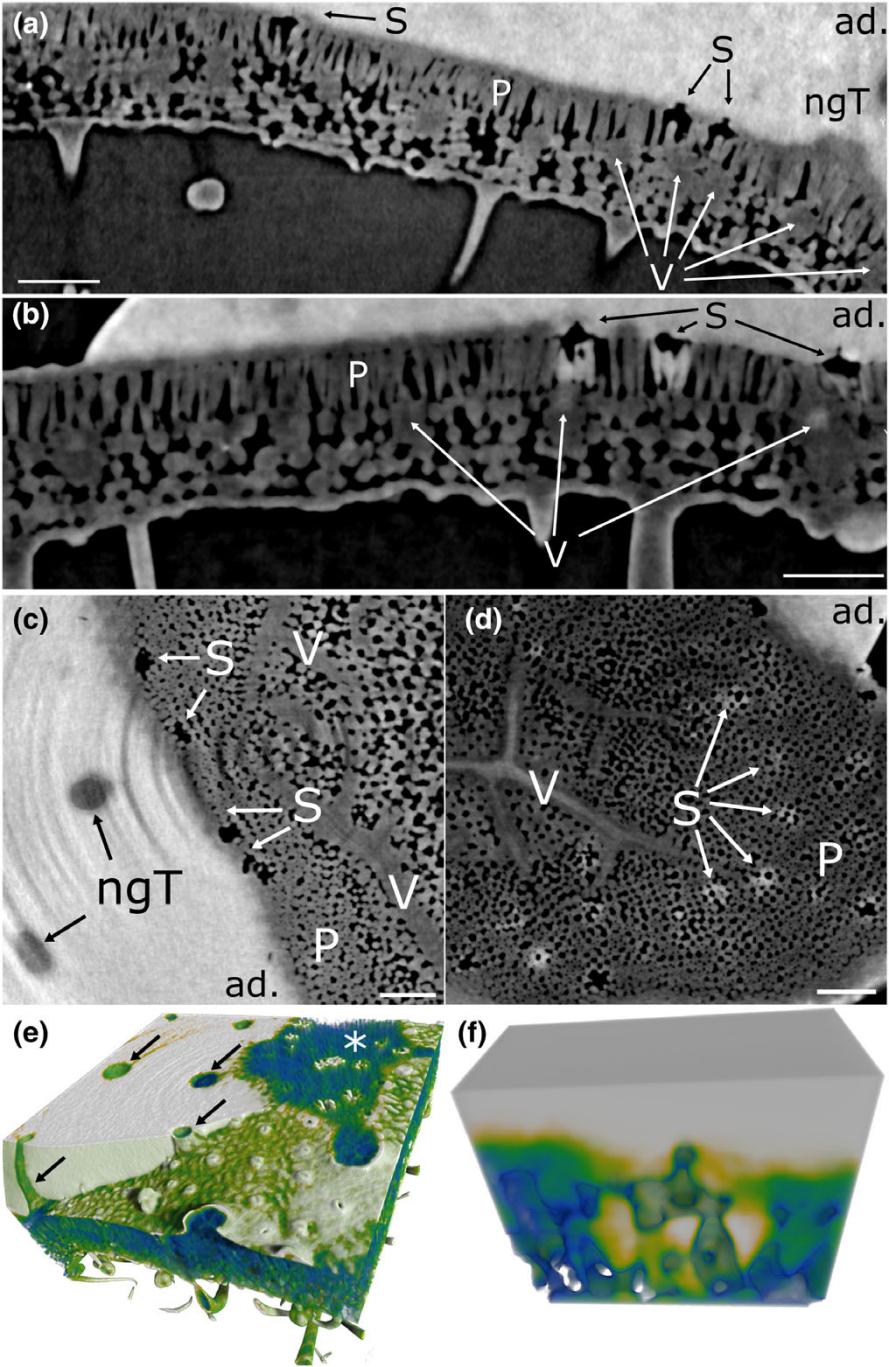
High surface tension droplets on a potato leaf. Virtual cross sections through a potato leaf with a water droplet deposited on the adaxial leaf side, imaged by X‐ray μCT. Bright areas correspond to water deposited on the leaf surface, gray areas correspond to leaf tissue, and dark areas correspond to air space. (a, c) No water uptake into the leaf is observed 25 min after droplet deposition. (b, d) After 7 h, water forms a continuum from the leaf surface into the substomatal cavities. Contrast agent translocates inside vascular bundles. (e) A 3D reconstruction shows that water from the droplet does not enter nonglandular trichomes (→), but sub‐stomatal cavities (*) after 7 h. (f) 3D reconstruction of a single hydraulically activated stoma fully covered by water after 7 h. (a, c) Vertical cross sections. (b, d) Horizontal cross sections. (e, f) 3D reconstructions, where the water film is depicted in white, while the leaf tissue appears blue/green. ab., abaxial leaf side; ad., adaxial leaf side; ngT, nonglandular trichome; P, palisade cells; S, stoma; V, vascular bundle. Bars, 100 μm.

Next, we applied droplets containing 150 mM iohexol as a contrast agent onto leaves. The liquid surface tension was first adjusted to γ = 70 mN m^−1^. It was found that the iohexol concentrations used did not affect the surface tension in comparison with milliQ water. Leaves with droplets or water films were repeatedly CT scanned after different time points up to 8 h after droplet deposition. Strong X‐ray absorption by iodine resulted in contrast between the water applied to the surface, the leaf tissue, and air space, allowing us to identify water originating from the treatment (Figs 4, 5).

On barley leaves, ridges with fiber cells and trichomes established physical contact with the water droplet within 20 min (Fig. 4a). By contrast, sunken stomata mostly failed to establish contact, even after 8 h (Fig. 4a,c; Video S3). Water was taken up exclusively across the sclerenchymatic ridges, while it was impossible to distinguish fiber cells from trichomes (Fig. 4b–d; Video S3). Water taken up along the bundle sheath extension entered the vasculature below by advancing along cells in the mesophyll (Fig. 4c; Video S3). From the entry points onwards, water was translocated inside veins. This is nicely shown by the bright contrast lines in Fig. 4(d) (annotation V).

Contrastingly, droplets deposited on potato leaves established physical contact with the whole surface within 25 min (Fig. 5a; Video S4). All adaxial surface structures were fully covered by water. At no point did we observe signs of cuticular water uptake through areas above the vasculature or fiber cells at trichome bases. Heads of glandular trichomes did not appear to take up any contrast agent (Videos S4–, S7). No stomata were penetrated by water initially (Fig. 5a,c; Video S4). After 3–5 h, more and more stomata became hydraulically activated (data not shown). Most stomata displayed hydraulic activation after 7 h (Fig. 5c,d; Video S5). Water entered the vasculature, within which it was translocated (Fig. 5c,d; Video S5). Direct connectivity between sub‐stomatal cavities and the vasculature was observed in some locations (Video S5). Based on an approximated resolution of 2 μm with the technique (3–4 pixels), the aqueous continuum connecting the droplet on the surface with the sub‐stomatal cavity appears to have an inhomogeneous thickness in the low μm range.

### Surface tension and topography determine leaf wetting

**Fig. 6:**
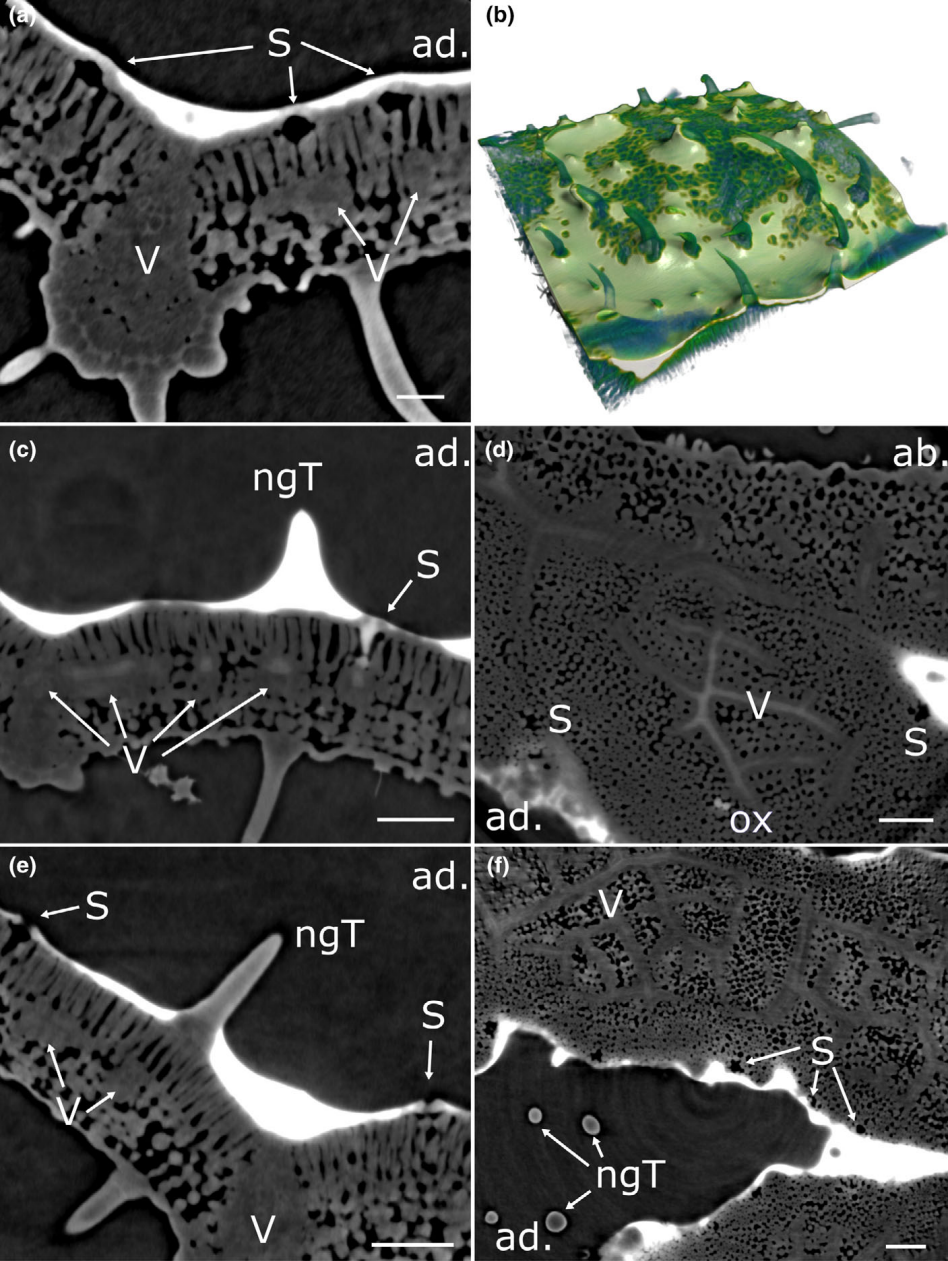
Liquid films develop from intermediate surface tension droplets on potato leaves. Virtual cross‐sections through a potato leaf with a water film at γ = 30 mN m^−1^ deposited on the adaxial leaf side and imaged by X‐ray μCT. Bright areas correspond to water deposited on the leaf surface, gray areas correspond to leaf tissue, and dark areas correspond to air space. (a, c, e) Vertical cross sections. (d, f) Horizontal cross sections. (b) 3D reconstruction where the water film is depicted in white and the plant tissue in blue/green. (a) No water uptake into the leaf is observed 15 min after droplet deposition. (b) A 3D model shows how water accumulates along veins and around trichomes. (c, d) After 5.5 h, hydraulic activation of stomata occurs in some of the stomata, and water is taken up into vascular bundles. (e, f) After 7.5 h, the water film becomes thinner, and water uptake decreases. ab., abaxial leaf side; ad., adaxial leaf side; ngT, nonglandular trichome; ox, oxalate crystal; S, stoma; V, vascular bundle. Bars, 100 μm.

Lowering the surface tension from 70 to 30 mN m^−1^ led to faster droplet spreading, resulting in a larger leaf area being wetted by a thinner water film. This results in water accumulation in topographically lower leaf areas, specifically along veins (Fig. 6a,b; Videos S6, S7). In addition, thicker water films spanned (stretched) between the cuticular leaf surface and tips of some nonglandular trichomes (Fig. 6b,c; Video S6), as well as in between single trichomes. Consequently, stomata located in proximity to veins or trichomes were often observed to be fully covered with water, while stomata located further away appeared to be covered to a lesser extent. Starting after 3 h, few stomata displayed HAS, and water entered the vasculature between 3 and 5.5 h after droplet application. Fig. 6(c,d) and Video S7 show the status after 5.5 h. After 8 h, the water film appeared overall thinner. Most stomata were no longer covered by it, and HAS became less evident. The signal coming from veins decreased (Fig. 6e,f). Again, no water uptake at trichome bases was observed.

**Fig. 7:**
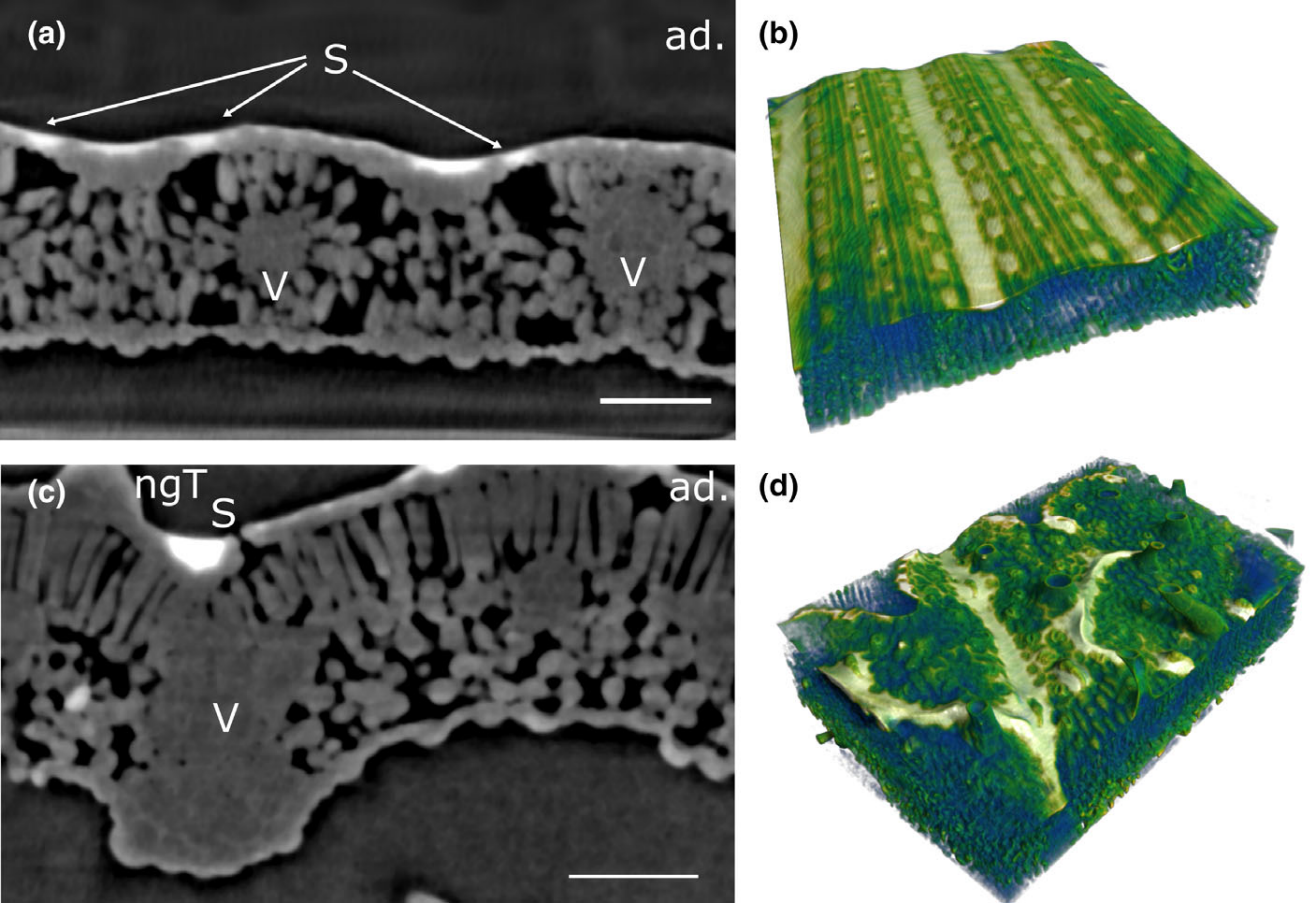
Liquid films from low surface tension droplets on leaves of barley and potato. Leaves were treated with droplets at γ = 20 mN m^−1^ and imaged by X‐ray μCT. (a, c) Vertical sections. (b, d) 3D reconstructions, where plant tissues are depicted in blue/green shades and the water film in white. (a, b) Barley leaf imaged 4.5 h after droplet deposition. Water accumulates as a thin film following the leaf topography, covering the leaf surface between veins and above sunken stomata. No penetration of stomata by water is observed. (c, d) Potato leaf 7.5 h after droplet deposition. The water film follows veins and is thicker around some trichome bases. The water film does not cover stomatal pores, and no hydraulic activation of stomata is seen. No uptake of contrast agent into vascular bundles is observed. ad., adaxial leaf side; ngT, nonglandular trichome; S, stoma; V, vascular bundle. Bars, 100 μm.

Further lowering of γ to 20 mN m^−1^ led to the fast creation of a thin water film of varying thickness both on barley and on potato leaves (Fig. 7). On barley, the whole leaf surface now established physical contact with the water film (Fig. 7a,b; Video S8). Sunken stomata formed small pools filled with water, and the film was thicker in areas on the leaf surface located between elevated veins. No HAS or cuticular uptake of water was observed at any time. Compared to treatments with higher γ, the thinner water film created less contrast and therefore made it challenging to identify uptake pathways. Contrast originating from the boundary between air‐filled substomatal cavities and mesophyll cells, as well as at the dense fibers at trichome bases, was indistinguishable from contrast agent when water films became thin. We estimate the resolution limit of the present technique to lie at *c*. 1.5–2 μm (3–4 pixels).

In contrast to barley, most raised stomata on potato leaves were no longer covered in water (Fig. 7c,d; Video S9). Instead, the water film followed topographic depressions of the leaf surface and accumulated at veins and in areas around trichome bases. Most elevations remained water‐free. Again, no water uptake could be identified at any time point, and no water uptake into the vasculature was observed.

## Discussion

### Novel insights into the stomatal pathway

It has been known for a long time that liquids with a surface tension above 30 mN m^−1^ fully covering a stoma were not able to penetrate stomatal pores due to their capillary resistance combined with stomatal hydrophobicity and geometry (Schönherr & Bukovac, 1972; Stevens, 1993). Later, Jürgen Burkhardt found that this capillary barrier can be overcome by the presence of hygroscopic aerosols undergoing drying and wetting cycles (Burkhardt, 2010; Burkhardt *et al*., 2012, 2018; Burkhardt & Hunsche, 2013; Basi *et al*., 2014), or under the presence of surfactants (Basi *et al*., 2014; Kaiser, 2014). The findings were explained by the fact that microscopic leaf wetness does not fully cover stomatal pores but rather follows the stomatal walls from the leaf surface into the apoplast (Burkhardt *et al*., 2012; Burkhardt & Hunsche, 2013). Here, we observe HAS on potato leaves in the absence of a surfactant (γ = 70 mN m^−1^) or any drying‐rewetting cycles in the presence of hygroscopic particles, indicating that HAS can occur to a strong degree, provided that the stoma is fully covered by a droplet of water (Fig. 5b,d,f). HAS has been estimated to occur as a < 100 nm thick water continuum that requires days to weeks to establish (Burkhardt *et al*., 2012, 2018; Vega *et al*., 2023), and only in a small fraction (< 10%) of all stomata on a leaf (Eichert *et al*., 2008; Eichert & Goldbach, 2008). However, in this study, we observe a μm‐thick water continuum penetrating most stomata under a droplet placed on a potato leaf within 7.5 h (Fig. 5b,d). Several factors might explain the discrepancy between literature and our findings. Firstly, evidence for HAS is based on studies from a few plant species, including leaves of hybrid poplar (Eichert & Goldbach, 2008; Vega *et al*., 2023), apple (Burkhardt *et al*., 2012), leek, faba bean, and coffee (Eichert *et al*., 2008; Eichert & Goldbach, 2008). That makes generalized conclusions challenging: for instance, we have time‐resolved information on HAS development based on whole leaf *g*_s_ measurements on hybrid poplar and confocal proof of an aqueous continuum across single stomata in apple leaves, but limited time resolution of the process. However, the differences between barley and potato plants observed here indicate that HAS might be as diverse as plants are: fully wetted open stomata on barley plants (γ = 20 mN m^−1^) do not appear activated, while fully wetted open stomata on potato leaves are (γ = 70 mN m^−1^). This might be due to differing surface chemistries (Fernández *et al*., 2017) in combination with different stomatal morphologies, for instance, the presence and shape of cuticular ledges on the guard cell surface (Eichert *et al*., 2008), or the presence of cuticular folds on the cell surface above and within the stoma (Pautov *et al*., 2019; Guzmán‐Delgado *et al*., 2021). The relative humidity within the stomatal pore under a water film is expected to prevail at 100%. Consequently, condensation of water on the cell surfaces within the pore can be expected. While condensation would occur in the form of droplets on a hydrophobic cell surface, condensation in the form of a thin film is to be expected on a more hydrophilic surface (e.g. Burkhardt & Hunsche, 2013). The difference in HAS formation between barley and potato leaves could thus be a reflection of the intra‐stomatal surface composition, where film condensation in stomata on potato leaves might favor HAS formation compared to droplet condensation in barley leaves. However, this remains speculative, as nanoscale surface imaging technologies such as tip‐enhanced Raman spectroscopy (TERS) and nano Fourier transform infrared spectroscopy (FTIR) on uneven leaf surfaces need further development before providing sound data on surface chemistry. Furthermore, the lack of comparative studies in different plant species might explain why the stomatal pathway was previously excluded as a possibility for droplets with high surface tensions, and why uptake of solutes and particles across stomata was observed in the absence of surfactants anyway (Burkhardt *et al*., 2012).

Secondly, most studies investigate HAS as a result of deliquescence of hygroscopic particles in the vicinity of stomata. These induce thin foliar water films called ‘microscopic leaf wetness’ on guard cell surfaces (Burkhardt & Hunsche, 2013). By contrast, we investigate HAS in response to droplet deposition. This resembles the deposition of raindrops, spray, or fog deposition events as investigated by Berry *et al*. (2014). [Correction added on 25 August 2025, after first online publication: the preceding sentence has been corrected.] The two processes might follow different timing (weeks vs hours) and differ in the induced water continuum thickness on guard cell surfaces (< 100 nm vs μm). Potentially, HAS induced by deliquescent particles forms a < 100 nm water continuum which could subsequently swell in the presence of foliar water originating from dewfall or rain events. Consequently, more emphasis should be put on distinguishing between different types of leaf wetness.

**Fig. 8:**
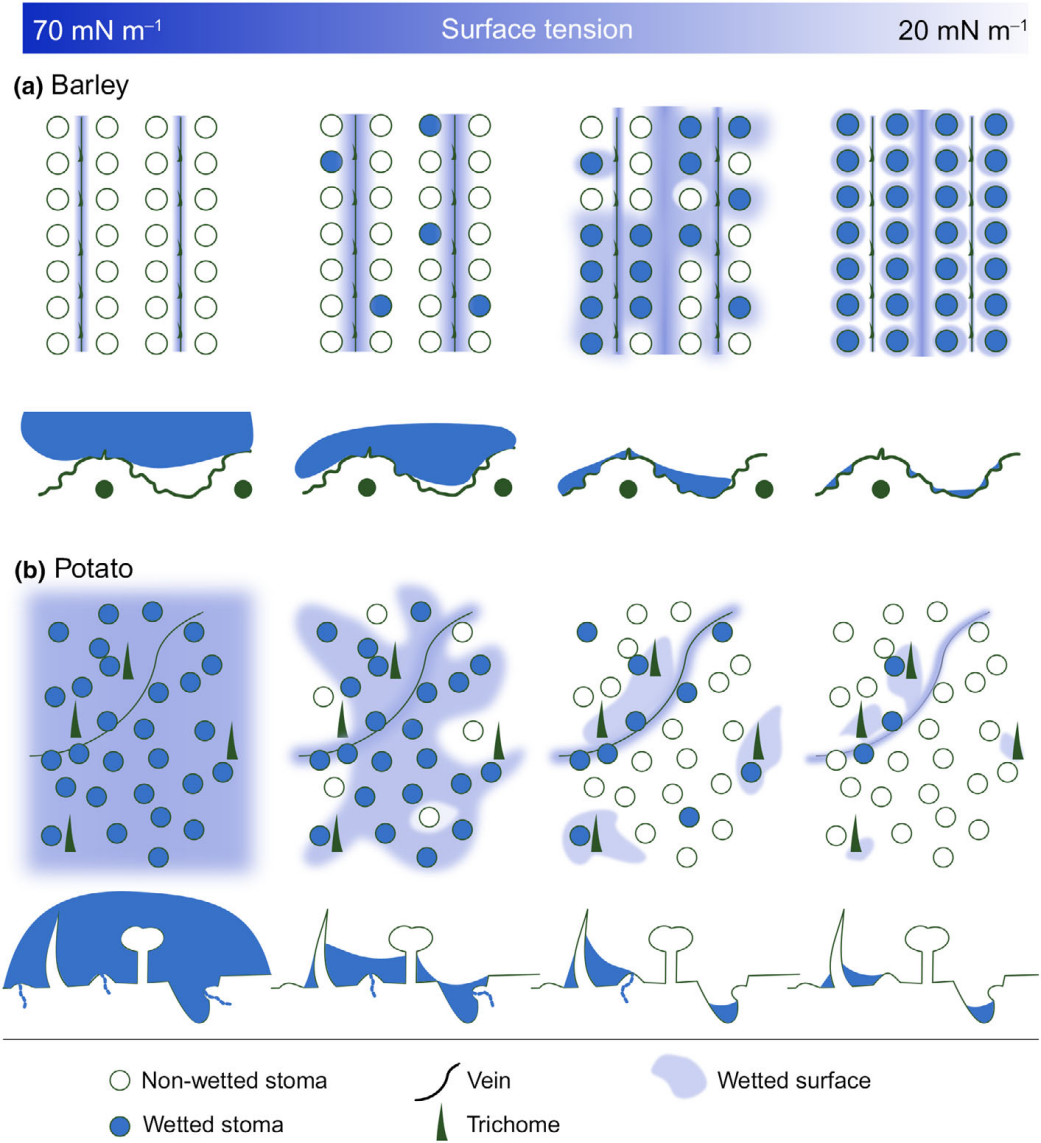
A model describing the wetting of stomata on leaves of potato and barley plants in relation to the liquid surface tension. The model shows top view and side view of barley (a) and potato (b) leaf surfaces. (a) On a barley leaf, a water droplet without surfactant (γ = 70 mN m^−1^) establishes contact exclusively with the sclerenchymal ridges above the vasculature, while stomata remain unwetted. As the surface tension decreases, the sclerenchymal ridges are gradually less wetted, while the topographical depressions, including sunken stomata and the region in between veins, are covered by a water film. At the lowest surface tension, γ = 20 mN m^−1^, all stomata are wetted, but none appear activated. (b) On a potato leaf, all surface features including stomata are in direct contact with a water droplet at γ = 70 mN m^−1^. As the surface tension decreases, water films span preferentially between trichomes and along veins, while elevated areas including raised stomata begin to remain unwetted. Therefore, stomata in proximity to trichomes and veins are preferentially wetted and activated compared to those located further away. At γ = 20 mN m^−1^, the water film becomes so thin that most stomata remain uncovered by the water film and are thus not activated.

Only < 10% of all stomata on a leaf surface were found to be activated in previous studies (Eichert & Burkhardt, 2001; Eichert *et al*., 2008; Eichert & Goldbach, 2008), which has lead to the assumption that only a fraction of all stomata on a leaf is generally hydraulically activated (Fernández *et al*., 2021; Husted *et al*., 2023). HAS appears to be an event occurring on the level of a single stoma (Grantz *et al*., 2018; Vega *et al*., 2023). It is described as a ‘random’ event, and it was hypothesized that this was due to the fact that stomatal guard cell surfaces have a history of aging, which results in priming of individual stomata for HAS (Burkhardt *et al*., 2018; Grantz *et al*., 2018; Vega *et al*., 2023). In other studies, HAS was excluded as a potential water uptake mechanism because the observed uptake rates were not high enough to be explained by transport along continuous water films. This indicated that ‘reverse transpiration’ occurred rather than HAS (Guzmán‐Delgado *et al*., 2021). However, this might also be explained by the hydraulic activation of only a small fraction of stomata at once. Our results highlight that the fraction of activated stomata depends on leaf topography, leaf wettability (illustrated in Fig. 8), and time. Only a few stomata on a potato leaf were activated at γ = 30 mN m^−1^, because raised stomata fell dry unless located at veins or close to trichomes (Figs 6, 8b). Trichomes on potato leaves might be water repellent (Papierowska *et al*., 2020), but once leaves were wetted, trichomes seemed to attract and maintain nearby water films (Figs 6c,e, 7e). Consequently, the small fraction of HAS reported previously can be viewed as a result of patchy surface wetting: on a potato leaf, HAS will more likely occur if a stoma is close to a vein, a trichome (Figs 6, 8b) – or, as in literature, a deliquescent particle. Therefore, in addition to priming of an individual stoma, the localization of a stoma relative to the foliar water film determines whether it will be wetted or not. Interestingly, the trend of stomatal wetting on barley leaves is different to that on potato leaves, but can also be interpreted as a randomized event (Figs 4, 7a,b, and illustrated in Fig. 8a). In a similar manner, HAS is time‐dependent: at γ = 30 mN m^−1^, HAS on a potato leaf appears to be little after 25 min, it peaks after 5 h, and declines after 7 h (Fig. 6). Therefore, the experimental time window is of great importance.

### The cuticular pathway

As elaborated on in the introduction, cuticle swelling and the formation of ‘transient hydrophilic pores’ under high relative humidity might explain most cuticular water uptake (Fernández *et al*., 2017, 2021; Tredenick *et al*., 2017; Kamtsikakis *et al*., 2021; Matos *et al*., 2024). Transient hydrophilic pores have an estimated size exclusion limit between 2 and 5 nm, but their existence remains to be confirmed experimentally (Eichert *et al*., 2008; Eichert & Goldbach, 2008; Fernández *et al*., 2016). Transport along the cuticular pathway has been related to leaf wetness. For instance, highly unwettable leaves of some grass species contain regions covered by water repellent nano‐structured epicuticular wax rodlets (Barthlott *et al*., 2017), while other areas might be more hydrophilic, allowing for direct contact with the surface liquid. Such water repellent structures might be missing on sclerenchymal ridges of barley leaves, allowing for contact with the droplet. Inhomogeneous cuticle wetting may lead to uptake rates that vary in space on a micron to submicron scale as a result of cuticle patchiness in terms of thickness, chemical composition, and nanoscale structure. A cuticle might even be fully absent around leaf structures such as trichomes or veins (Fernández *et al*., 2017, 2024). The different surface wettability of barley and potato leaves (Fig. 1) also underlines the importance of surface chemistry when it comes to cuticle wetting. However, while nano‐spectroscopic imaging methods are emerging, the techniques still need significant development. Therefore, nanoscale physical and chemical heterogeneity of barley and potato leaf cuticles remain largely unknown. Our data underline that leaf topography and surface tension are relevant factors determining the location and persistence of leaf wetness, and therefore the activation of the cuticular pathway. Relatively high liquid surface tensions (γ = 70 mN m^−1^) on a barley leaf allow direct contact with the topographic ridges rich in fiber cells. Within 3 h after droplet deposition, surface water appears to start penetrating across the cuticle into the bundle sheath extension (data not shown). Within 7 h after droplet deposition, the water enters the vasculature, from where it is translocated along the leaf axis (Fig. 4a,b). By contrast, water films at low surface tension (γ = 20 mN m^−1^) fail to fully cover the sclerenchymal ridge and might dry out faster because of their reduced thickness (Fig. 7). It should be noted that a high concentration of surfactant might plasticize or disintegrate the cuticle and increase its porosity, thereby giving rise to cuticular uptake (Knoche *et al.*, 1992). However, most uptake observed here occurred in the absence of a surfactant (γ = 70 mN m^−1^). Rather than disintegration of the cuticle, the extent of water uptake along this pathway seems to be dependent on direct contact between water and leaf, which is a result of surface tension and leaf topography, and possibly chemical properties. These areas of contact are required to be chemically prone to swelling for FWU to occur.

Generally, these results are in line with findings from Arsic *et al*. (2020), where phosphate ions traveled along fiber cells into the vasculature of barley plants. The observed uptake has been attributed to swelling of pectin‐rich cell walls (Arsic *et al*., 2020). Here, the contrast agent iohexol, which is a highly soluble and ionic molecule with a nonhydrated diameter of 1.0–1.7 nm used as an apoplastic marker (Cheng *et al*., 2022; Gao *et al*., 2023), was able to cross the cuticle. This indicates that potential transient hydrophilic pores in barley cuticles must have at least this diameter. We found no sign of water penetration across the cuticle in the region between veins, indicating the absence of such hydrophilic pores in this region (Figs 4, 7), or the failure to establish contact between water and cuticle due to localized water repellency. Similarly, we did not observe cuticular water uptake on potato leaves, neither at veins nor at trichome bases (Figs 5, 6, 7).

Further improvements to the imaging technology include better contrast without increasing sample exposure times and tissue damage. These approaches are discussed in the ‘Potentials and limitations of X‐ray μCT imaging’ section. Such developments might allow investigation of cuticle and cell wall swelling, and to better distinguish between fiber cells and trichomes on sclerenchymal ridges of barley leaves. These would also allow to study water uptake across trichome bases of potato leaves, which is suggested to follow similar mechanisms as cuticular uptake and is widely understudied (Schreel *et al*., 2020; Li *et al*., 2023). With the current setup, we might overlook FWU when occurring in smaller quantities and over longer time periods as a result of current restrictions in terms of resolution and contrast.

### What happens after leaf surface penetration?

Both in barley and potato plants, we observe that the contrast agent enters the vasculature a few hours after droplet deposition (Figs 4b–d, 5b,d). The direct flow of water from the entrance point toward the vasculature indicates the presence of either a water potential or an osmotic potential (Schreel *et al*., 2019; Schreel & Steppe, 2020; Coopman *et al*., 2021) rather than diffusion as a driving force. Plants have been placed in nutrient solution within a humid atmosphere (> 90% RH) over several hours, making a water potential gradient as a driver of FWU improbable (Schreel & Steppe, 2020). Rather, such a direct targeting of the contrast agent toward the vasculature might be a result of local osmolyte accumulation next to the vasculature, resulting in an osmotic attraction of surface water (Schenk *et al*., 2021). Due to the μm scale restrictions in resolution power, it remains a subject of further investigation whether iohexol entered the xylem or phloem or just stayed in the apoplast of cambium/parenchyma within the vascular bundle. However, in previous studies, iohexol has been shown to be an apoplastic marker that is unable to penetrate plasma membranes (Gao *et al*., 2023).

### Potentials and limitations of X‐ray μCT imaging

The scanning protocol presented in this work was developed and optimized for a minimum of X‐ray dose while acquiring data of sufficient quality. Utilizing the phase contrast available at modern synchrotrons is a major advantage compared with classical absorption CT (Zambelli *et al*., 2010; Lovric *et al*., 2013; Spiecker *et al*., 2023). The presented data demonstrate the ability to keep plants alive for extended periods (> 8 h) and withstand multiple X‐ray CT scans (up to 14 tomograms) without visible beam damage. Dose sensitivity was primarily assessed through visual inspection of the scan quality after multiple scans of an untreated control. The results indicate that leaf cells remain at full turgor, and all stomata are open, suggesting that the plants remain healthy and maintain photosynthetic activity. An open question is whether prolonged X‐ray exposure could cause long‐term damage that manifests over several days. While this is not a concern for the investigation of HAS due to its short timescales, it may become a limitation for future long‐term experiments (Montanha *et al*., 2023). Additionally, the sample holder used in this study is designed for young plants to accommodate beamline space constraints. Scaling up to larger plants would pose significant challenges in most beamline setups and could degrade image quality due to increased propagation distances and local tomography artifacts from larger samples. One potential solution is transitioning to multi‐distance phase contrast methods, but this would substantially increase scan times, raising the risk of sample movement during scanning (Martínez‐Criado *et al*., 2016). It should be noted that X‐ray imaging of leaves can be improved by laminography, where leaves are placed vertically rather than horizontally (Verboven *et al*., 2015). However, this was not possible here because aqueous solutions would have run off the surface.

One challenge of the method is that thin, μm‐scale water films create little attenuation‐based contrast compared to the phase contrast originating from phase boundaries, such as the leaf surface or the cell surfaces in the sub‐stomatal cavity. Edge contrast effects can be observed in the absence of a water film, which were our control treatments (Fig. 3). This makes it difficult to identify small amounts of contrast agent present along boundaries between plant cells and air. Future setups could include utilizing the full phase contrast available at fourth generation synchrotrons with holotomography setups (Martínez‐Criado *et al*., 2016) or even single distance approaches using coded apertures for maximum phase contrast with minimum X‐ray dose (Nikitin *et al*., 2025). Such methods would allow for advanced image segmentation procedures and to draw quantitative conclusions based on the local density of materials, even at the nanoscale. Algebraic and deep learning reconstruction methods could reduce the needed number of projections, ultimately reducing scanning durations and radiation damage to the sample (Piovesan *et al*., 2021; Villarraga‐Gómez *et al*., 2022; Jørgensen *et al*., 2023).

In the given case, we do not observe any HAS in the stomata of barley, even if open stomata are fully covered by liquid over hours. We might conclude that HAS does not occur, but the contrast agent originating from a < 100 nm thin water continuum would not create resolvable contrast compared to the phase contrast between water‐filled mesophyll cells and the sub‐stomatal cavity. Water penetration might only become visible if the contrast agent stained parenchymal cells or entered the vasculature, which would create contrast relative to nonexposed parenchymal tissue in the neighborhood. Improved contrast and resolution, advanced post‐processing, contrast thresholding, and segmentation might allow us to enhance our understanding of cuticle and fiber cell swelling, as well as loading into the vasculature.

### How can we complement X‐ray μCT?

While X‐ray μCT can help to address various questions about FWU, complementing it with other techniques will allow for a more in‐depth understanding of the underlying mechanisms. For instance, cuticular water entry points observed in X‐ray CT could be spatially correlated with chemical surface composition or cuticle thickness by the use of atomic force microscopy with a TERS probe (Fernández *et al*., 2024), FTIR, or confocal RAMAN imaging (Heredia‐Guerrero *et al*., 2014). Morphological knowledge obtained from TEM will simplify the interpretation of X‐ray CT data (Fernández *et al*., 2016; Guzmán‐Delgado *et al*., 2021). Simultaneously, *post hoc* analyses with samples from X‐ray CT may allow for the recognition of artifacts, provide chemical information, and improve data interpretation. Such techniques include X‐ray fluorescence microscopy (Du *et al*., 2015) and laser ablation inductively coupled plasma mass spectrometry (LA‐ICP‐MS) (Arsic *et al*., 2020). For imaging FWU, magnetic resonance imaging, positron emission tomography, and neutron imaging can be complementary, but remain to be improved in resolution, and in some cases in scanning time (Dhondt *et al*., 2010; Cai *et al*., 2022; Blystone *et al*., 2024). So far, the visualization of FWU mechanisms is dependent on tracer molecules or ions, which are inherently different from the water molecule itself. However, especially the fact that heavy water (D_2_O) creates contrast relative to water (H_2_O) in neutron imaging bears the potential for *in vivo* imaging of foliar water films in the absence of chemically distinct tracers.

### Concluding remarks and outlook

In order to study foliar water films *in vivo*, we have developed an X‐ray μCT based method that allows 3D imaging of plants growing in a controlled environment. Contrast and resolution were improved to a level which allows acquisition of data in good quality, while structural radiation damage was limited by reduced exposure times and optimization of energy. This experimental setup will enable FWU studies in a variety of plant species, with varying water and nutritional status, age, or when exposed to stress. It will not only allow studying the effect of surfactants of FWU, but also that of hygroscopic particles, humectants, fungal hyphae, or other components of the phyllosphere. In this study, we show how leaf topography and liquid surface tension determine the location of contact between water and the leaf surface by controlling water film formation. Because leaf topography and wettability differ between plant species, the conditions that activate either the cuticular or the stomatal pathway differ as well. While cuticular FWU occurs across pectin‐rich fiber cells in barley leaves, HAS seems to be the major pathway for FWU in potato leaves. We provide the first experimental evidence of a water continuum connecting the leaf surface with mesophyll cells via the sub‐stomatal cavity. In some species and under the right conditions, HAS can occur within hours in the absence of surfactants or hygroscopic particles, in quantities beyond what has been reported before. Besides providing novel mechanistic information, further method development will enable FWU quantification, which can be complementary to stable isotope techniques. We expect that this will be useful in a variety of related research fields, especially when studying foliar pathogen infection and uptake of deposited airborne particles, and not least in the development of more efficient fertilizers and agrochemicals.

## Competing interests

None declared.

## Author contributions

SH, MF and EVK designed the experiment. IE and AS cultivated plants, performed gas exchange measurements, and together with MF, EVK, FS and SH developed the sample holder to allow μCT scanning in a controlled atmosphere. RD and KGD performed optical tensiometry and the epidermal imprints. MF, EVK, FS and AS collected μCT data. MF, EVK and RM reconstructed and processed CT data, and with SH, they performed interpretation. MF, EVM and SH drafted the paper, with contributions from AS. All authors approved the final manuscript. MF and EVK contributed equally to this work.

## Disclaimer

The New Phytologist Foundation remains neutral with regard to jurisdictional claims in maps and in any institutional affiliations.

## Supporting information


**Fig. S1** Relationship between liquid surface tension and Silwet Gold surfactant concentration.
**Fig. S2** Stomatal conductance of potato leaves under varying light intensities and CO_2_ concentrations.
**Fig. S3** Light microscopy images of epidermal leaf imprints of potato and barley leaves showing stomatal aperture and closure under controlled conditions.
**Fig. S4** 3D reconstructions of barley leaves obtained by X‐ray micro‐CT showing aperture and closure of stomata by controlling the surrounding atmosphere and light conditions.
**Fig. S5** 3D reconstructions of potato leaves obtained by X‐ray micro‐CT showing aperture and closure of stomata by controlling the surrounding atmosphere and light conditions.
**Fig. S6** Percentage of open stomata and their average aperture width for barley and potato leaves in an uncontrolled vs a controlled atmosphere based on 3D reconstructions from X‐ray micro‐CT.

**Video S1** Barley control without droplet.

**Video S2** Potato control without droplet.

**Video S3** Barley, droplet with γ = 70 mN m^−1^, after 8 h.

**Video S4** Potato, droplet with γ = 70 mN m^−1^, after 25 min.

**Video S5** Potato, droplet with γ = 70 mN m^−1^, after 7 h.

**Video S6** Potato, droplet with γ = 30 mN m^−1^, after 5.5 h, A.

**Video S7** Potato, droplet with γ = 30 mN m^−1^, after 5.5 h, B.

**Video S8** Barley, droplet with γ = 20 mN m^−1^, after 5 h.

**Video S9** Potato, droplet with γ = 20 mN m^−1^, after 7.5 h.
Please note: Wiley is not responsible for the content or functionality of any Supporting Information supplied by the authors. Any queries (other than missing material) should be directed to the *New Phytologist* Central Office.

## Data Availability

The reconstructed μCT images and raw projection images are available as .tiff files at doi: 10.11583/DTU.28235483. 3D visualization videos of all CT images presented in the paper can be accessed at doi: 10.11583/DTU.28645601.
