# Supplementary material for: X‐ray micro‐CT imaging to study foliar water uptake mechanisms in plants with contrasting leaf topography
Source: New Phytol. 2025 Aug 9;248(2):656–71. doi: 10.1111/nph.70421 (PMC12445830; doi:10.1111/nph.70421)
Supplement: Supplementary file 1 — Fig. S1 Relationship between liquid surface tension and Silwet Gold surfactant concentration. Fig. S2 Stomatal conductance of potato leaves under varying light intensities and CO2 concentrations. Fig. S3 Light microscopy images of epidermal leaf imprints of potato and barley leaves showing stomatal aperture and closure under controlled conditions. Fig. S4 3D reconstructions of barley leaves obtained by X‐ray micro‐CT showing aperture and closure of stomata by controlling the surrounding atmosphere and light conditions. Fig. S5 3D reconstructions of potato leaves obtained by X‐ray micro‐CT showing aperture and closure of stomata by controlling the surrounding atmosphere and light conditions. Fig. S6 Percentage of open stomata and their average aperture width for barley and potato leaves in an uncontrolled vs a controlled atmosphere based on 3D reconstructions from X‐ray micro‐CT. [file NPH-248-656-s004.pdf]

New Phytologist

***New Phytologist* Supporting Information**

Article title: X-ray micro-CT imaging to study foliar water uptake mechanisms in plants with contrasting leaf topography
Authors: Max Frank, Emil Visby Kristensen, Augusta Szameitat, Francesca Siracusa, Idil Ertem, Rikke Dahl, Katie G. Dempsey, Rajmund Mokso, Søren Husted
Article acceptance date: 07 July 2025

The following Supporting Information is available for this article:

Fig. S1 Relationship between liquid surface tension and Silwet Gold surfactant concentration.

Fig. S2 Stomatal conductance of potato leaves under varying light intensities and CO2 concentrations.

Fig. S3 Light microscopy images of epidermal leaf imprints of potato and barley leaves showing stomatal aperture and closure under controlled conditions.

Fig. S4 3-D reconstructions of barley leaves obtained by X-ray micro-CT showing aperture and closure of stomata by controlling the surrounding atmosphere and light conditions.

Fig. S5 3-D reconstructions of potato leaves obtained by X-ray micro-CT showing aperture and closure of stomata by controlling the surrounding atmosphere and light conditions.

Fig. S6 Percentage of open stomata and their average aperture width for barley and potato leaves in an uncontrolled versus a controlled atmosphere based on 3-D reconstructions from X-ray micro-CT.

Video S1 barley control without droplet

Video S2 potato control without droplet

Video S3 barley, droplet with $\gamma = 70$ mN m^{-1}, after 8 h

Video S4 potato, droplet with $\gamma = 70$ mN m^{-1}, after 25 min

Video S5 potato, droplet with γ = 70 mN m^{-1}, after 7 h

Video S6 potato, droplet with γ = 30 mN m^{-1}, after 5.5 h, A

Video S7 potato, droplet with γ = 30 mN m^{-1}, after 5.5 h, B

Video S8 barley, droplet with γ = 20 mN m^{-1}, after 5 h

Video S9 potato, droplet with γ = 20 mN m^{-1}, after 7,5 h

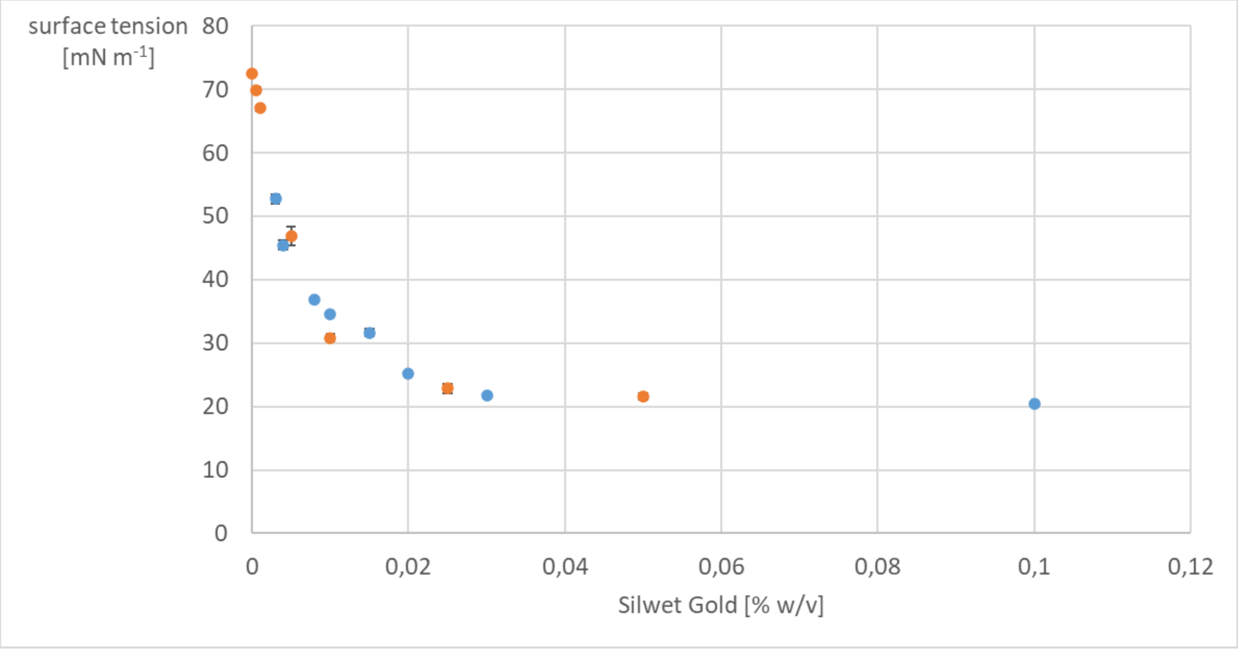

Fig. S1 Relationship between liquid surface tension and Silwet Gold surfactant concentration in milliQ water (orange) and in a 150 mM iohexol solution (blue) measured with optical tensiometry. Error bars show standard deviations, one confidence interval, n = 5.

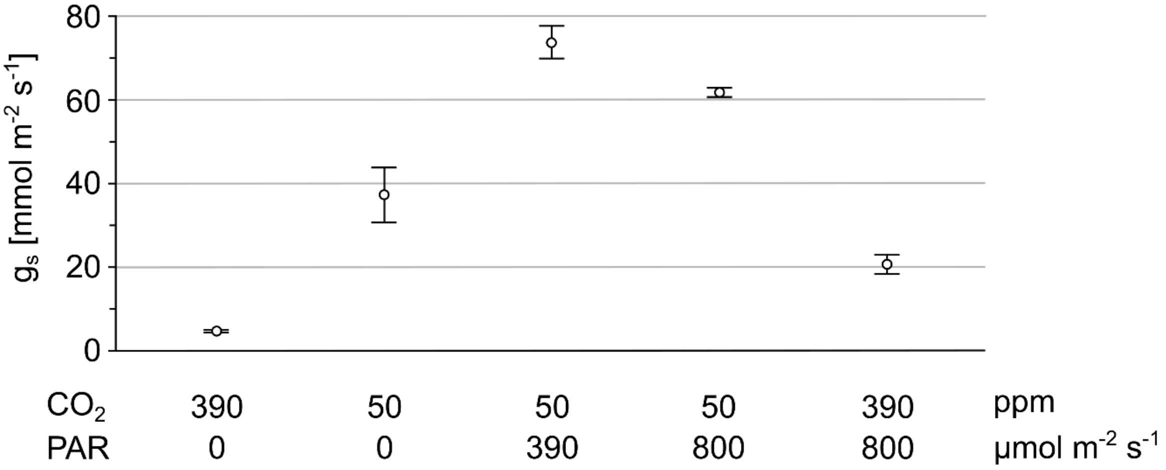

Fig. S2 Stomatal conductance gs of potato leaves measured with a leaf cuvette of CIRAS 3 under the indicated light conditions and varying atmospheric concentrations of CO2. Plants were exposed to light and atmosphere for 30 min prior to data acquisition. Error bars show standard deviations of 5 technical replicates on youngest fully evolved leaves of 3 independent plants (n = 15).

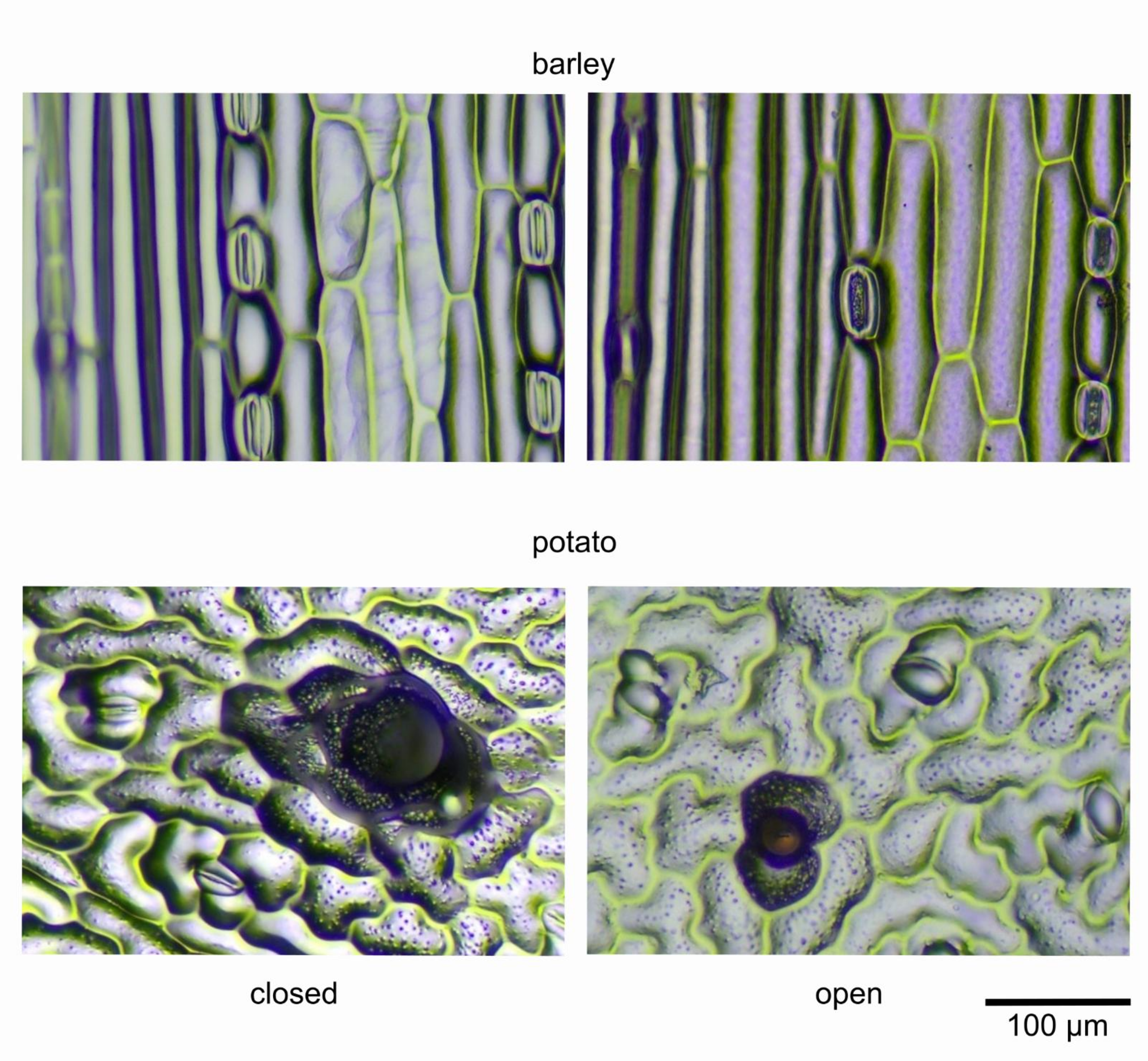

Fig. S3 Light microscopic images of adaxial leaf epidermal imprints of youngest fully evolved leaves of barley and potato. The images display the successful control of stomatal aperture and closure after 30 min incubation time. Closed: Ambient CO_2 and darkness. Open: <50 ppm CO_2 and 390 µmol $m^{-2}s^{-1}$.

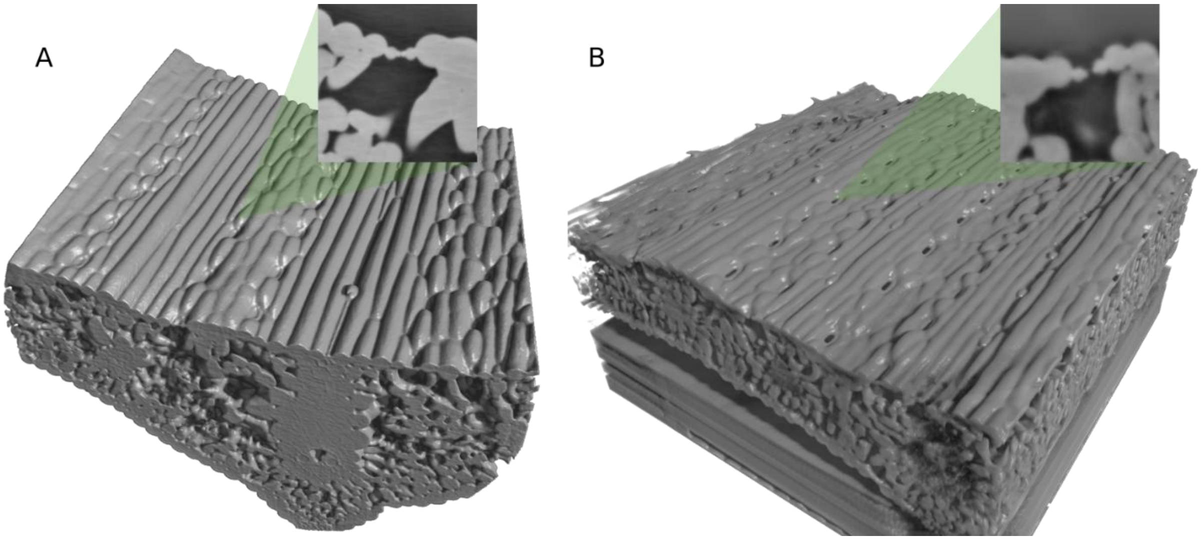

Fig. S4 X-ray µCT reconstructions of untreated barley leaves. (**a**) Barley leaf exposed to ambient laboratory air and light prior to scanning. Most stomata on the adaxial side are closed. (**b**) Barley leaf in a controlled atmosphere containing <50 ppm CO2 at >90 % RH. All stomata are significantly more open than observed in (**a**). Both plants have been placed under growth light in vermiculite soaked in nutrient solution.

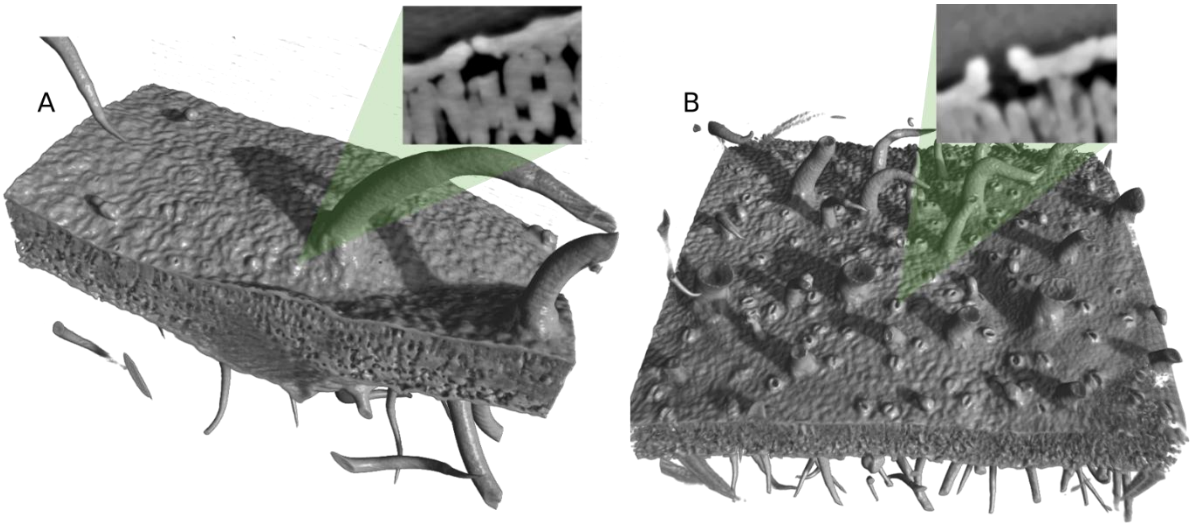

Fig. S5 X-ray µCT reconstructions of untreated potato leaves. (**a**) Potato leaf exposed to the ambient laboratory air and light before scanning. (**b**) Potato leaf under controlled atmosphere containing <50 ppm CO2 and >90% RH under growth light. The controlled atmosphere is seen to have the effect of opening most stomata, which tend to topographically elevate above the

leaf surface. Both plants have been placed under growth light in vermiculite soaked in nutrient solution.

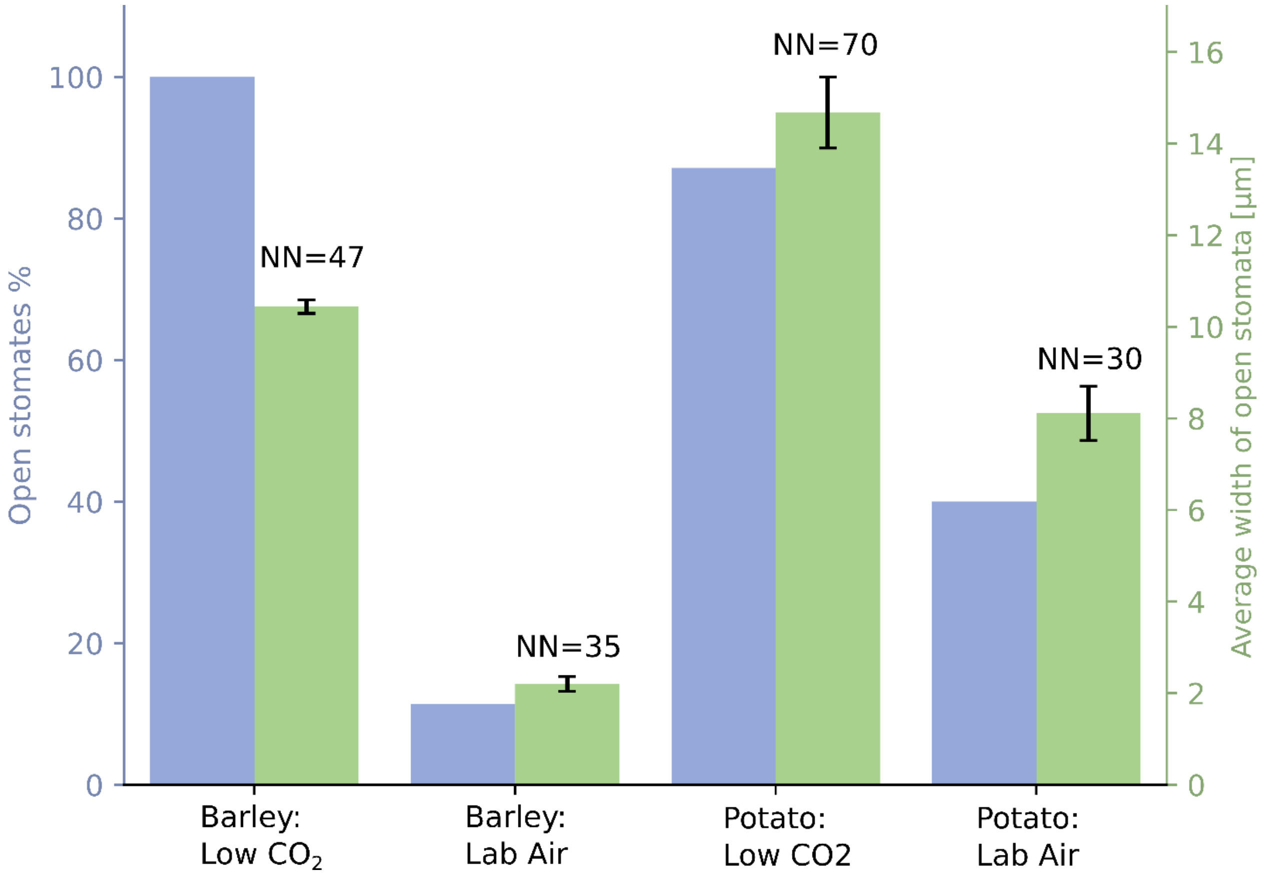

Fig. S6 Aperture of stomata identified through µCT. In blue: percentage of stomata that are identified as being open. In green: average aperture width of the open stomata in the field of view while disregarding closed stomata. low CO_2: <50 ppm CO_2, 390 mmol m^{-2} light, > 90 % RH. Lab Air: non-controlled environment at the DanMAX beamline at MaxIV in Lund, SE. Error bars show standard deviations.

Videos S1-S9 are available here: DOI: 10.11583/DTU.28645601

Video S1: 3D visualization of a barley leaf from *in vivo* X-ray µCT. All stomata on the adaxial leaf side are open, and positioned in parallel lines between veins. Veins appear as elevated sclerenchymal ridges, with hook-shaped non-glandular trichomes. By contrast, stomata are sunken structures, resembling tiny wells.

Video S2: 3D visualization of a potato leaf from *in vivo* X-ray µCT. All stomata on the adaxial leaf side are open, are unevenly distributed. Veins appear as depressions, and both long non-glandular trichomes and smaller glandular trichomes are distributed on the leaf surface. Stomata are raised structures.

Video S3: 3D visualization of a $\gamma = 70$ mN m^{-1} droplet on a barley leaf 8h after application imaged by *in vivo* X-ray µCT. Most sclerenchymal ridges establish direct contact with the overlying droplet, while sunken stomata mostly fail to do so. Iohexol from the droplet enters the leaf across the cuticle in areas above veins, while no hydraulic activation of stomata is observed. Water from the surface subsequently enters the vasculature, from where it is translocated further.

Video S4: 3D visualization of a $\gamma = 70$ mN m^{-1} droplet on a potato leaf 25 min after application imaged by *in vivo* X-ray µCT. The droplet establishes contact with all surface structures of the leaf, therefore fully covering trichomes and stomata below it. At this time point, no hydraulic activation of stomata is observed yet.

Video S5: 3D visualization of a $\gamma = 70$ mN m^{-1} droplet on a potato leaf 7 h after application imaged by *in vivo* X-ray µCT. The droplet is still present and in physical contact with all surface structures of the leaf. A some µm thick water continuum spans from the leaf surface into the sub-stomatal cavity of most stomata located under the overlying droplet. By contrast, water does not appear to enter the leaf across the cuticle or at trichome bases.

Video S6: 3D visualization of a $\gamma = 30$ mN m^{-1} water film on a potato leaf 5.5 h after application

imaged by *in vivo* X-ray µCT. The droplet has spread out into a water film of varying thickness. Water accumulates close to trichomes and in the topographic depressions (i.e. along the veins). Simultaneously, only few of the elevated stomata are covered by the liquid film. The closer the stoma is located to a vein or a trichome, the higher appears the chance to be wetted by the overlying film.

Video S7: 3D visualization of a γ = 30 mN m^{-1} water film on a potato leaf 5.5 h after application imaged by *in vivo* X-ray µCT. This video shows the same sample as video S6. Hydraulic activation only occurs in the few stomata that are still covered by the water film, and the water appears to enter the vasculature. Most stomata, which are raised above the surface, are no longer covered by the thin water film and are thus not hydraulically activated.

Video S8: 3D visualization of a γ = 20 mN m^{-1} water film on a barley leaf 5 h after application imaged by *in vivo* X-ray µCT. The initial droplet has fully spread into a thin film. Water accumulates in the lower regions of the leaf (i.e. above sunken stomata and in the parallel lines between veins), while raised leaf areas are more prone to falling dry. Although all stomata are wetted and open, no hydraulic activation of stomata can be observed. At the same time, no water uptake along sclerenchymal ridges above veins can be seen, either.

Video S9: 3D visualization of a γ = 20 mN m^{-1} water film on a potato leaf 7.5 h after application imaged by *in vivo* X-ray µCT. The initial droplet has fully spread into a thin film of varying thickness. Water accumulates in the lower regions of the leaf (i.e. above veins and close to some of the trichomes), while raised leaf areas such as stomata are falling dry. No foliar water uptake is observed under these conditions.
